# Globotriaosylceramide Gb3 Influences Wound Healing and Scar Formation by Orchestrating Fibroblast Heterogeneity

**DOI:** 10.1002/advs.202509733

**Published:** 2025-08-14

**Authors:** Sujie Xie, Runzhi Huang, Weijin Qian, Xinran Ding, Wei Zhang, Yixu Li, Jianyu Lu, Hanlin Sun, Yifan Liu, Yuntao Yao, Bingnan Lu, Minjuan Wu, Zhaofan Xia, Shizhao Ji

**Affiliations:** ^1^ Department of Burn Surgery The First Affiliated Hospital of Naval Medical University Shanghai 200433 China; ^2^ Department of Ophthalmology Shanghai Ninth People's Hospital Shanghai Jiao Tong University School of Medicine Shanghai 200011 China; ^3^ Department of Urology Xinhua Hospital Affiliated to Shanghai Jiao Tong University School of Medicine Shanghai 200092 China; ^4^ BGI Research BGI‐Hangzhou Hangzhou 310012 China; ^5^ Department of Histology and Embryology Naval Medical University Shanghai 200433 China

**Keywords:** fibroblast heterogeneity, globotriaosylceramide, hexosaminidase subunit beta, scar formation, wound healing

## Abstract

Cutaneous fibroblast heterogeneity is mechanistically linked to wound repair outcomes and fibrotic progression, with glycosphingolipid metabolism emerging as a critical determinant of physiological fibroblast diversity. Through integrative analysis of spatiotemporal omics, lipidomics, and single‐cell RNA sequencing (scRNA‐seq) coupled with histological evaluation of clinical specimens, the functional involvement of globotriaosylceramide (Gb3) in dermal regeneration processes is systematically investigated. Comparative profiling reveals significant upregulation of Gb3 biosynthesis in superficial second‐degree burns (SSDB) relative to deep second‐degree burn (DSDB) injuries. Hexosaminidase subunit beta (HEXB) is identified as the exclusive differentially expressed Gb3 synthase distinguishing these injury subtypes. Functional validation through in vitro and in vivo models demonstrates that pharmacological suppression of HEXB‐mediated Gb3 synthesis exacerbates fibroblast‐to‐myofibroblast transdifferentiation, attenuated fibroblast growth factor 2 (FGF2) signal transduction, and ultimately potentiated fibrotic scarring. These findings establish a novel HEXB‐Gb3‐FGF2 regulatory axis governing fibroblast phenotypic plasticity in differential‐depth skin injuries, providing mechanistic insights for developing targeted antifibrotic therapies.

## Graphical abstract

In superficial second‐degree burn wounds, Gb3 turns on genes related to papillary cells through the FGF2 signaling pathway. This increases the ability of cells to break down fibrin and decreases fibrosis, which ultimately prevents scar formation in burn injuries. The deep second‐degree burn wounds augment the fibrotic capacity of the cells, resulting in the development of hypertrophic scars. Created in BioRender.

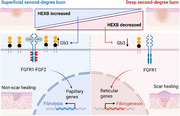

## 1 Introduction

Burn injuries constitute a major global health burden, with recent WHO data (2023) documenting an annual mortality rate exceeding 180 000 cases (https://www.who.int/news‐room/fact‐sheets/detail/burns). Among the categorizations based on depth and size, different thickness dermal injuries, especially second‐degree burn (SDB), stand out as the most prevalent among outpatients.^[^ ^1^ ^]^ This type of burn injury is further classified into superficial SDB (SSDB), involving only the papillary layer of the dermis, and deep SDB (DSDB), which extends into both the papillary and reticular layers of the dermis.^[^ ^2^ ^]^ Multiple inflammatory pathways are activated, driving fibroblasts to transdifferentiate into myofibroblasts. DSDBs frequently progress to pathological wound healing with hypertrophic scarring. Conversely, SSDB tends to have less severe outcomes. Hypertrophic scar formation manifests as pain, itch, contractures, limb stiffness, and neuropathic pain, which are detrimental to patients’ appearance and sensorimotor function, decreasing their quality of life. It also hinders patient rehabilitation and delays social reintegration.^[^ ^3^ ^]^ In DSDB, fibroblasts in the reticular layer are affected more prominently, while their heterogeneous nature might contribute to the varying outcomes in scar formation between DSDB and SSDB.^[^ ^4^ ^]^ As the currently studied mechanism of scar formation after SDB remains unclear, it is imperative to investigate the distinct functional properties and underlying molecular mechanisms of fibroblasts postburn injury. Such investigations hold profound implications for the development of targeted scar intervention therapies.

Lipid metabolism is prevalent in keloid formations, such as high levels of arachidonic acid are a prominent characteristic of keloids.^[^ ^5^ ^]^ In addition to being key components of the skin, lipids serve as sources of secondary messengers, such as ceramide, which can influence cellular processes involved in keloid formation.^[^ ^6^ ^]^ Glycosphingolipids (GSLs) are essential components of biological membranes, consisting of a ceramide backbone connected to a glycan moiety. Due to the abundance of glycan headgroups, GSLs can be divided into ganglio‐series (GM/GT/GD), globo‐series (Gb), lacto‐series (LC), and asialo‐series (GA).^[^ ^7^ ^]^ A study using pseudotime trajectory analysis proposed that the increment of GSLs metabolism pathway activity is correlated with fibroblast differentiation.^[^ ^8^ ^]^ In one of the latest articles, Laura Capolupo et al. made a significant elucidation of Gb synthesis's influence on dermal fibroblast heterogeneity.^[^ ^9^ ^]^ They discovered that fibroblast identity is determined in part by the Gb or GM enriched on its membrane through the fibroblast growth factor 2 (FGF2) signaling pathway. According to previous discoveries, its activation fosters Gb synthesis by α 1,4‐galactosyltransferase (A4GALT) expression in normal human skin and stimulates fibroblasts to transform into the papillary subtype with better fibrolytic as well as proliferative capabilities.^[^ ^10^ ^]^ Based on the above studies, we speculate that the differences in Gb metabolism between DSDB and SSDB may affect the characteristics of fibroblasts, which may underline the divergent healing outcomes of DSDB and SSDB. As there are many Gb synthesis‐related genes (GbRGs), such as *A4GALT*, ST3 β‐galactoside α‐2,3‐sialyltransferase 1 (*ST3GAL1*), hexosaminidase subunit α (*HEXA*), and hexosaminidase subunit β (*HEXB*), we aimed to illuminate the key molecules involved in the Gb‐fibroblast differentiation process in SDBs, so as to offer novel ideas for preventing and treating burn scars.

Recent advancements in high‐resolution sequencing technologies and experimental validation have demonstrated that dermal fibroblasts can be classified into papillary and reticular subpopulations, each characterized by distinct biological traits and spatial distribution.^[^ ^11^ ^]^ Papillary fibroblasts are responsible for hair follicle formation and re‐epithelialization of wound healing, while reticular fibroblasts are mainly involved in mediating fibrogenic fibrous extracellular matrix (ECM) remodeling as well as dermal structure renovation. Although these innovative technologies have provided details of gene expression and fibroblast characteristics in human skin, the limited resolution and lack of spatial information greatly hindered an elaborate distinction of fibroblasts in the two layers of dermis, as well as the understanding of their distribution and morphological identities.^[^ ^12^ ^]^ Here, we applied a remarkable method called spatially enhanced resolution omics‐sequencing (Stereo‐seq) with single cell resolution and high sensitivity to determine spatially resolved single‐cell transcriptomes of human skin SDB sections, which helped to identify intricate key molecules’ spatial location, fibroblast characteristics in different layers, and development of wound healing processes.^[^ ^12^ ^]^ In this study, single‐cell RNA sequencing (scRNA‐seq), spatial transcriptomics (ST), Stereo‐seq, and lipidomics were performed on the SDB samples from our burn patients, suggesting differential expression of globotriaosylceramide (Gb3) between SSDB and DSDB. In view of the differential expression of Gb3 and the different healing outcomes of superficial and deep second‐degree burns, we hypothesized that Gb3 may play a protective role in burn wounds. The expression of Gb3 in SSDB was higher than that in DSDB. It also identified a key GbRG, *HEXB*, which was enriched in fibroblasts with a proliferative phenotype and was positively correlated with the FGF2 signaling pathway. After validation by cell experiments, the crucial regulatory axis HEXB‐FGF2 was constructed, demonstrating higher expression in SSDB and promoting Gb synthesis and fibroblast functional transformation to papillary state. Gb3 has demonstrated efficacy in reducing scar formation postburn in vivo. Taken together, we reveal the transformational role of Gb3 in fibroblast heterogeneity and demonstrate its potential as a therapeutic target for the treatment of burn wounds. Our data is expected to provide potential therapeutic targets for the management of scars after SDB with extraordinary innovation and significance.

## 2 Results

### 2.1 Gb Expresses Differentially Between DSDB and SSDB

**Figure 1:**
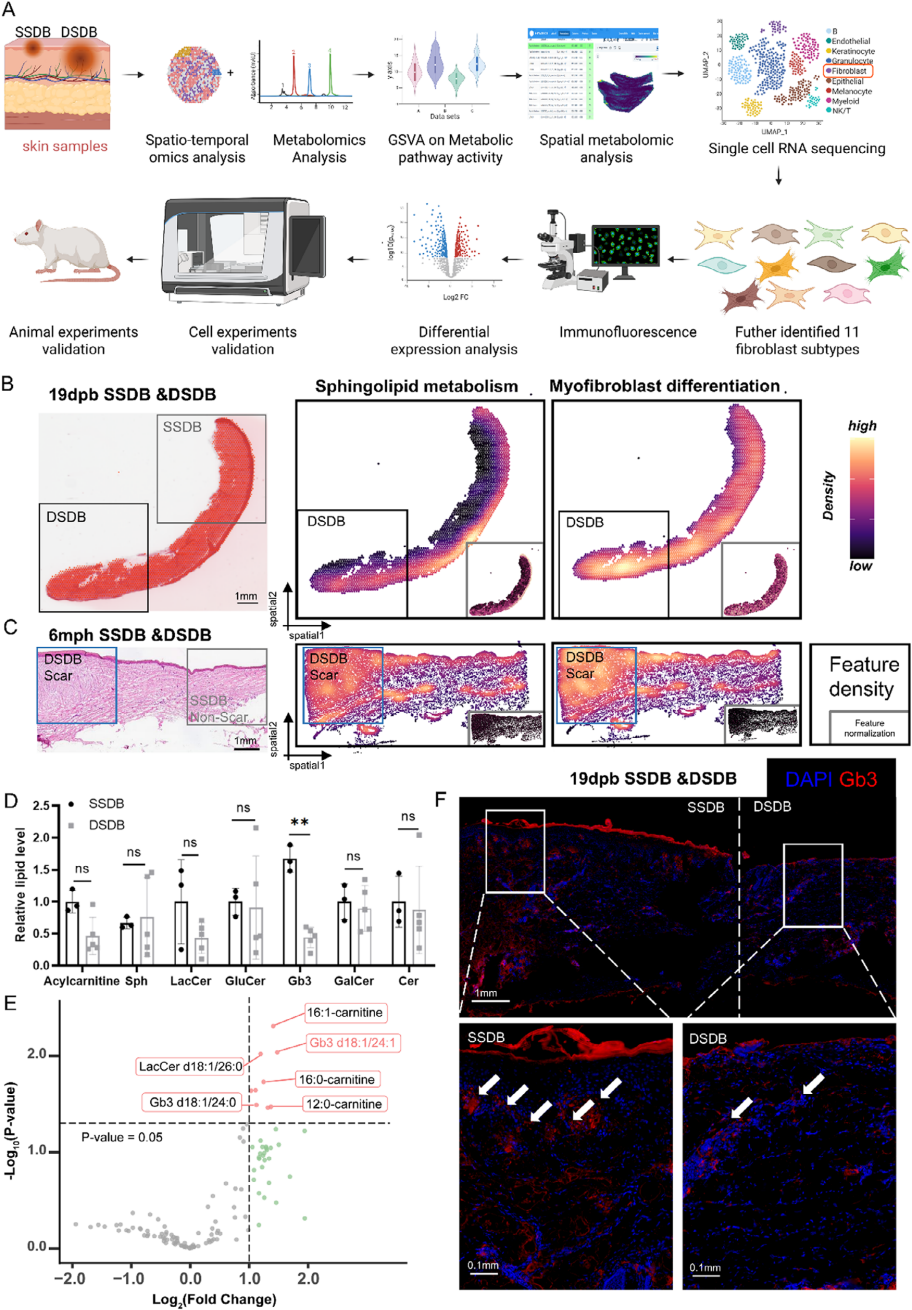
Combination of ST and metabolomics analysis revealed differentially expressed Gb3 between DSDB and SSDB. A) Study flow chart of Gb3 for scarless burn wound healing. Created in BioRender. B,C) Spatiotemporal Transcriptomic Analysis of Burn Wound Samples. Left panel: Hematoxylin and eosin (H&E) staining. Middle panel: Enrichment map for Sphingolipid Metabolism pathway. Right panel: Enrichment map for Myofibroblast Differentiation pathway. For each enrichment map (middle and right), the main panel displays raw expression values, while the inset in the lower‐right corner shows normalized expression values. B) Mixed superficial and deep second‐degree burn sample at postburn day 19. The region highlighted by the black box in the lower‐left corner of the H&E image represents the deep second‐degree burn (DSDB) area. Within both the Sphingolipid Metabolism and Myofibroblast Differentiation enrichment maps, the DSDB region exhibits a more yellowish hue, indicating higher expression levels of pathway‐associated genes compared to surrounding regions. C) Mixed superficial and deep second‐degree burn sample at postburn month 6. The region highlighted by the blue box on the left side of the H&E image represents the DSDB scar area. In both the Sphingolipid Metabolism and Myofibroblast Differentiation enrichment maps, the DSDB scar region shows a more yellowish coloration, signifying elevated expression levels of pathway‐associated genes relative to adjacent areas. D,E) Lipid metabolism suggested that Gb3 in lipids was significantly increased in SSDB (*n* = 3). The data are presented as the means ± SDs. **p < 0.01. F) Immunofluorescence suggested that the expression of Gb3 was higher in SSDB than that in DSDB.

The experimental workflow is schematized in **Figure** **1**A; and Figure S1 (Supporting Information). As an initial investigative approach, ST was employed to assess depth‐dependent variations in Gb metabolism between DSDB and SSDB. Due to the different depths of the wounds involved in DSDB and SSDB, the required time for epithelialization had a discrepancy, which resulted in different epithelium morphology of the two SDBs at the same time. Figure 1B depicts a representative burn specimen at 19 days postburn (dpb), with histologically confirmed SSDB (gray‐demarcated region) demonstrating complete re‐epithelialization in the upper quadrant, contrasting with persistent epithelial defects in the DSDB zone (black‐demarcated lower quadrant). It was then submitted to the ST analysis platform and generated a spatially mapped gene expression profile. Gene Set Variation Analysis (GSVA) was implemented for spatial visualization of the metabolic pathways’ activity, with black to yellow indicating elevated gene expression intensity. It was discovered that the sphingolipid metabolism was significantly different in its spatial distribution and intensity of related genes in DSDB region was higher than that in SSDB region. We also analyzed burn scar samples and peri‐scar skin 6 months posthealing (mph) after SSDB and DSDB. The results showed that sphingolipid metabolism was significantly increased in the DSDB‐scar region (shown in blue frame), and this region also had high expression of myofibroblast differentiation genes (Figure 1C). The other samples at different time points also showed a correlation between sphingolipid metabolism and both wound repair and scarring (Figure S2A, Supporting Information).

To systematically investigate sphingolipid metabolic alterations in SDB, targeted polar lipid metabolomic profiling was performed on SSDB and DSDB patient skin samples using normal‐phase high‐performance liquid chromatography (NP‐HPLC). The clinical characteristics of patients with SSDB and DSDB are shown in Table S1 (Supporting Information). All participants were middle‐aged males (age range: 28–47 years; mean±SD: 36.50±6.35 years), eliminating confounding effects from gender and minimizing age‐related metabolic variability in this cohort. There were no significant outliers in the lipid sequencing principal component score map (Figure S2B, Supporting Information), and the heat map showed significant differences in Gb3 on SSDB and DSDB (Figure S2C, Supporting Information). Further analysis of the data (volcano plot and bar plot) confirmed that Gb3 levels were significantly increased in SSDB (Figure 1D,E). Next, immunofluorescence staining for Gb3 was performed in burn wounds and scar skin. Gb3 levels were found to be significantly reduced in DSDB wounds compared with SSDB wounds (Figure 1F; and Figure S2D, Supporting Information). These findings collectively demonstrate depth‐dependent Gb3 dysregulation mechanistically linked to wound pathophysiology, with SSDB exhibiting enhanced sphingolipid metabolic activity that may confer protective effects during epithelial regeneration.

### 2.2 Mainly Distribution of Gb in Dermal Papillary Fibroblasts

**Figure 2:**
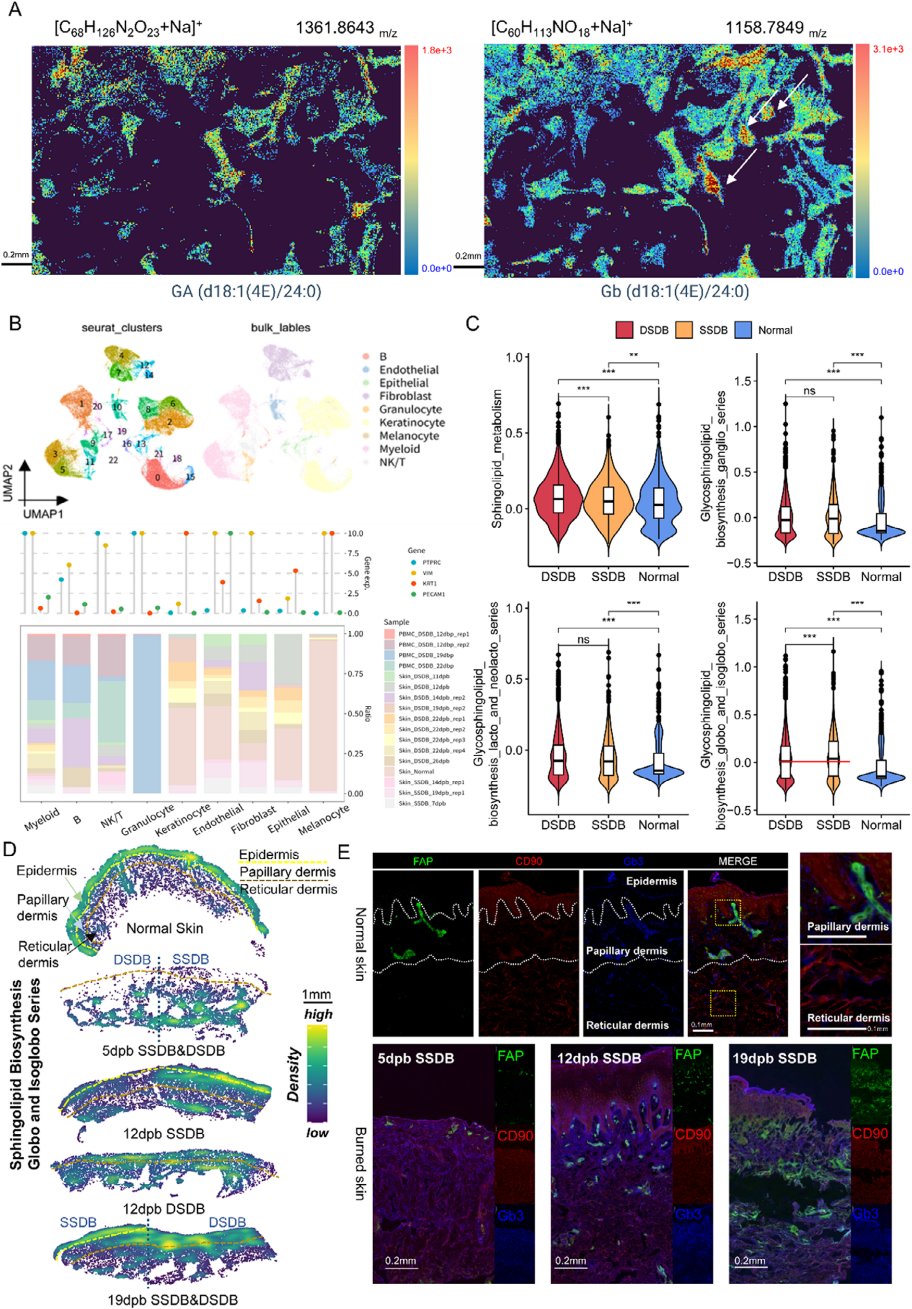
Gb differentially located in dermal fibroblasts and mainly distributed in papillary instead of reticular fibroblasts. A) Spatial metabolomics analysis of normal skin displayed that Gb predominantly lay in small, spindle‐shaped papillary fibroblasts rather than large, stellate reticular fibroblasts, with spatial statistical significance. According to cytotoxin staining in a previous study, it was deciphered that Gb enriched in papillary fibroblasts. B) The scRNA‐seq analysis of burned and normal skin samples identified 9 cell clusters and the Cleveland plot showed cell types distribution in distinct samples as well as the accuracy of cell annotation with four canonical markers (PTPRC, VIM, KRT1, and PECAM1). C) GSVA quantitively identified that the activity of Gb synthesis metabolic pathway was significantly stronger in SSDB than that in DSDB, while other types of GSL born no significance. The data are presented as the means ± SDs. **p < 0.01, ***p < 0.001. D) Stereo‐seq analysis revealed predominant enrichment of the Gb metabolic pathway within the papillary dermis. The region superficial to the yellow dotted line denotes the epidermis, while the zone bounded by the yellow and brown dotted lines corresponds to the papillary dermis. The compartment deep to the brown dotted line represents the reticular dermis. Color intensity within each anatomical region indicates pathway expression levels. The lighter the color, the higher the expression level. E) Immunofluorescence verified that Gb mainly existed in papillary fibroblasts in both normal and burned skin samples.

Spatial metabolomics was performed to map the distribution of target metabolites in tissue samples. In **Figure** **2**A, the color gradient from blue to red corresponds to increasing abundance of the target metabolite. The abundance of GA was compared with Gb in the same normal skin tissue. Gb was predominantly localized in small, spindle‐shaped papillary fibroblasts (white arrows) compared to large, stellate reticular fibroblasts, showing statistically significant spatial localization. This finding aligns with previous cytochemical staining results from human skin biopsies, which demonstrated predominant Gb localization in the papillary dermal layer and its enrichment in papillary fibroblasts.^[^ ^9^ ^]^

On account of the dispersed involvement of dermal layers, the SDB sample provided an outstanding model to study dermal fibroblast heterogeneity. The SSDB wound retained a portion of the papillary layer along with the complete reticular layer, whereas the DSDB wound retained only fragmented remnants of the reticular layer. Therefore, we conducted scRNA‐seq to characterize Gb expression patterns within SDB fibroblasts.

The detailed original scRNA‐seq data came from the clinical samples of our burn patients. After strict quality control (QC), normalization, principal components analysis (PCA), and uniform manifold approximation and projection (UMAP) analysis, samples were clustered into 23 subpopulations, which were defined as 9 cell subtypes and annotated as B cell, endothelial cell, epithelial cell, fibroblast, granulocyte, keratinocyte, melanocyte, myeloid cell, and NK/T cell (Figure 2B). The typical feature plots of marker genes for each subtype, intuitively displayed the distribution of each cluster in the UMAP plot (Figure S3A, Supporting Information). The Cleveland histogram exhibited the distribution proportion of all samples and expression levels of 4 classical marker genes (*PTPRC*, *VIM*, *KRT1*, and *PECAM1*) in the previously identified 9 clusters (Figure 2B). Since there were representative markers for specific cell types, such as *PTPRC* for immunocytes, *VIM* for fibroblasts, *KRT1* for keratinocytes, and *PECAM1* for vascular endothelial cells, the cell annotation results were verified. Moreover, among PBMC and skin samples, only B cells, granulocytes, myeloid cells, and NK/T cells were recognized in PBMC samples, and other skin samples mainly consisted of fibroblasts and keratinocytes, which were indeed populated respectively in the dermis and epidermis layers of the skin (Figure S3B, Supporting Information). Cell cycle analysis in Figure S3C (Supporting Information) showed that the G2M‐ and S‐phase cells accounted for a large proportion of the fibroblast, keratinocyte, and NK/T cell clusters, indicating that these 3 cell subtypes possessed a vigorous proliferative character in SDBs. The significantly up‐ or down‐regulated differentially expressed genes (DEGs) among all 23 clusters were demonstrated in Figure S3D (Supporting Information). The certain gene was designated as a highly up‐regulated DEG when the average log_2_ Foldchange was greater than 0.5 and the adjusted *p*‐value was less than 0.01. In addition to the dot plot, the top 5 expressed DEGs in 9 clusters were pictured in a heat map, further validating the cell annotation results (Figure S3E, Supporting Information).

To elucidate the Gb metabolism profile in DSDB, SSDB, and normal skin at single cell level, we performed a quantitative metabolic analysis to determine the differential activities of 49 metabolic pathways (Figure S4A,B, Supporting Information). The activity of Gb biosynthesis metabolism pathway differed markedly between SSDB and DSDB, while GM and LC showed no significance between the two grades of SDB (Figure 2C). In contrast to Gb biosynthesis, the median activity of the sphingolipid metabolism pathway was stronger in DSDB than in SSDB, which might be partly attributed to the biosynthesis and transition process of GSL from sphingolipid.^[^ ^13^ ^]^ Moreover, in order to identify the precise orientation of Gb in SDB clinical samples, a technique with higher spatial resolution called Stereo‐seq was conducted (Figure 2D). The color from black to yellow indicates an increase in gene expression. Gb biosynthesis mainly enriched in the epidermis and papillary dermis in normal skin, while in SDB samples, it displayed a gradual increment over time in papillary dermis, with slightly higher density in SSDB than DSDB (see dotted lines for the distinction between epidermis, papillary dermis, and reticular dermis). By immunofluorescence staining, we also demonstrated that Gb was mainly expressed in papillary fibroblasts. The papillary and reticular layers of the dermis were indicated using FAP and CD90.^[^ ^14^ ^]^ CD90 exhibited strong expression in fibroblasts located in the lower dermis, but was rarely observed within the superficial dermis. In contrast, FAP showed high expression in fibroblasts within the papillary dermis. Additionally, there was a stronger costaining of Globotriaosylceramide (Gb3) with FAP, which was evident in both normal and burned skin (Figure 2E).

As cell communication analysis demonstrated, fibroblasts had a complex interaction network with other eight cell types as well as a self‐regulating personality (Figure S4C,D, Supporting Information). Owning to this result and the combination of indispensable functions of fibroblasts in burn wound healing and skin structure reestablishment, the fibroblast cluster was isolated to explore Gb's underlying mechanism in SDB. We first investigated its effect on dermal human fibroblast (dHFB) from normal skin. The scRNA‐seq data of five differently treated dHFB groups were downloaded from the aforementioned article^[^ ^9^ ^]^ and were performed as routine analysis procedures. A total of six fibroblast clusters were identified (Figure S4E, Supporting Information) with characteristic markers verification (Figure S4F,G, Supporting Information). In Figure S4H (Supporting Information), the proportion of C‐X‐C motif chemokine ligand (CXCL) fibroblast subtypes dominated in OEGb4S group apparently and exhibited great difference from other treatment groups. The OEGM3S group consisted of a majority of smooth muscle Actin Alpha 2 (ACTA2) fibroblast subtype, related to fibrogenesis phenotype of reticular fibroblast.^[^ ^14^ ^]^ It was speculated that Gb expression would contribute to fibroblast heterogeneity in normal skin while Gb and GM might exert an opposite function. Therefore, it was an interesting issue to understand Gb's role and its molecular mechanism in fibroblast identity in SDB.

### 2.3 Gb Synthesis‐Related Gene HEXB Differs in Dermis of DSDB and SSDB with Temporal Variation

**Figure 3:**
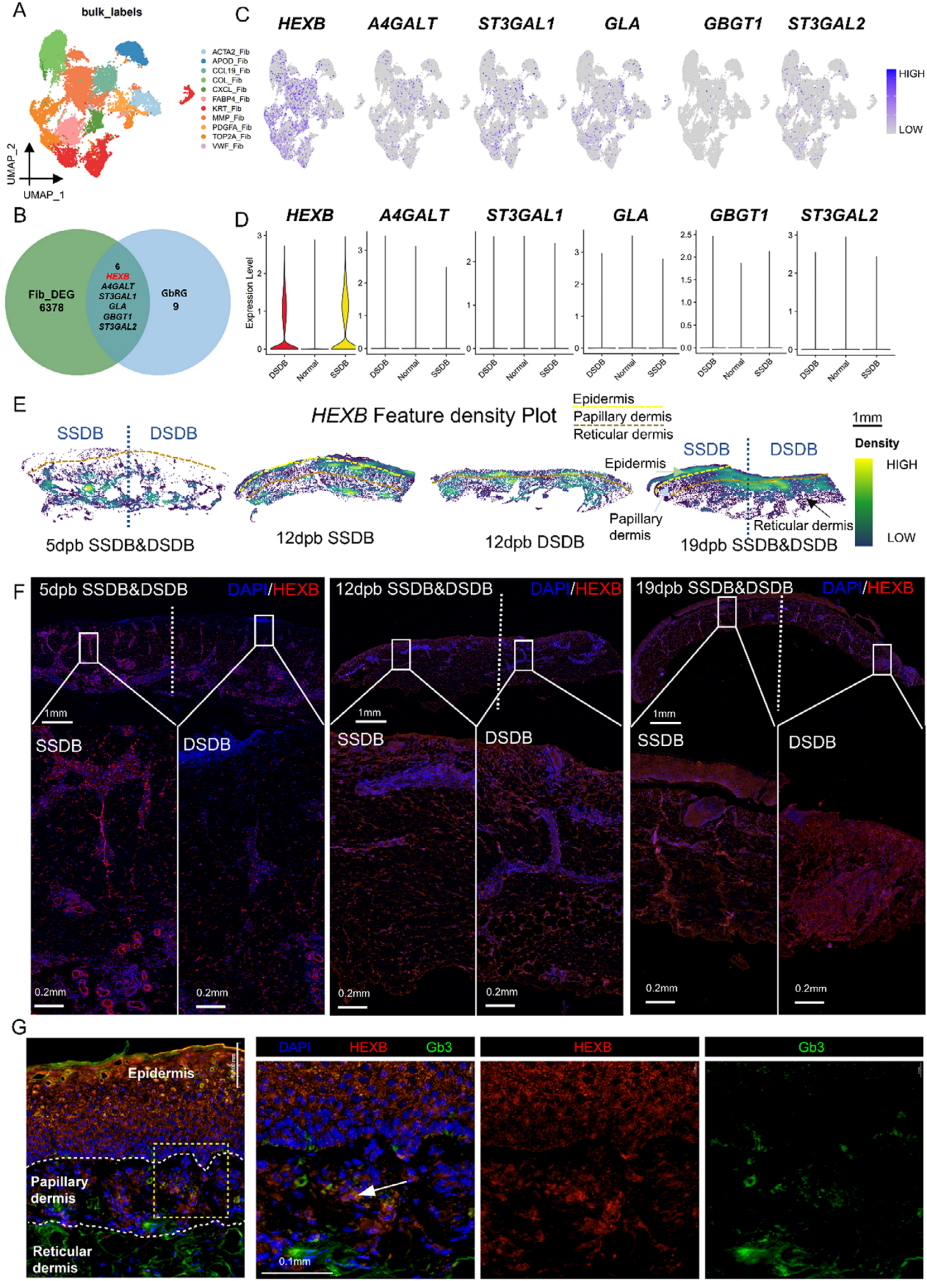
HEXB differentially expressed in dermal of DSDB and SSDB with temporal variation. A) scRNA‐seq of fibroblasts in burned skin samples identified 11 subtypes. B) Intersection of GbRGs and DEGs of fibroblasts in DSDB and SSDB showed 6 significant GbRGs (HEXB, A4GALT, ST3GAL1, GLA, GBGT1, ST3GALT2). C,D) Cellular feature plots and violin plots of the 6 GbRGs indicated that HEXB had the most significant difference between DSDB and SSDB. E) Stereo‐seq elucidated that HEXB was mainly expressed in papillary dermis and varied with the development of wound healing. The region superior to the yellow dashed line denotes the epidermal layer, while the area delimited between the yellow dashed line and brown dashed line corresponds to the papillary dermis. The compartment inferior to the brown dashed line represents the reticular dermis. Color intensity within each anatomical region indicates HEXB expression levels. The lighter the color, the higher the expression level. F) Immunofluorescence revealed temporal variation of HEXB expression. G) Immunofluorescence found that Gb3 (green) and HEXB (red) costained cells (orange) existed in papillary dermis of burned skin samples.

We subsequently extracted fibroblasts from the 9 previously identified cell types in our scRNA‐seq analysis to further investigate the regulatory mechanism of Gb in fibroblasts during SDB. A repeated scRNA‐seq analysis procedure was performed, identifying 11 fibroblast subclusters (**Figure** **3**A). We detected 6378 DEGs in fibroblasts between DSDB and SSDB groups and cross‐referenced these with 15 GbRGs, yielding six overlapping differentially expressed GbRGs, including *HEXB*, *A4GALT*, *ST3GAL1*, galactosidase α (*GLA*), globoside α‐1,3‐*N*‐acetylgalactosaminyltransferase 1 (*GBGT1*), and *ST3GAL2* (Figure 3B). The feature plots and violin plots of the 6 GbRGs (Figure 3C,D), indicating that HEXB was the most differentially expressed between DSDB and SSDB, compared to the other 5 GbRGs. HEXB is the beta subunit of the lysosomal enzyme beta‐hexosaminidase that can catalyze Gb3 synthesis.^[^ ^15^ ^]^ Stereo‐seq deciphered that the density of HEXB was principally higher in the papillary dermis of SDB, which increased progressively over time postburn (Figure 3E). This observation was subsequently validated through immunofluorescent staining.

Immunofluorescence analysis revealed dynamic temporal alterations in HEXB expression within dermal compartments between SSDB and DSDB cohorts. During the inflammatory stage (5 dpb), SSDB samples exhibited high expression of HEXB, whereas DSDB samples displayed low expression. As the wound progressed through healing stages, marked by fibroblast proliferation (12 dpb) and remodeling (19 dpb), DSDB samples exhibited higher levels of HEXB expression compared to SSDB (Figure 3F). Concurrently, our analysis revealed that most of the cells colocalizing with HEXB and Gb3 in burn samples were primarily located in papillary fibroblasts, aligning with our previous conclusions (Figure 3G). Consequently, we suggested that HEXB, an enzyme involved in Gb production, was a crucial factor influencing fibroblast identity in SDB.

### 2.4 Alteration of HEXB Results in Codirectional Alterations of Gb3 in Fibroblasts Affecting Cell Heterogeneity

Reticular fibroblasts are known to have more cells expressing the myofibroblast marker *ACTA2*, and are mainly in a fibrogenic state. Conversely, papillary fibroblasts demonstrate heightened proliferative capacity and are predominantly associated with basal, inflammatory, and fibrolytic states.^[^ ^16^ ^]^

**Figure 4:**
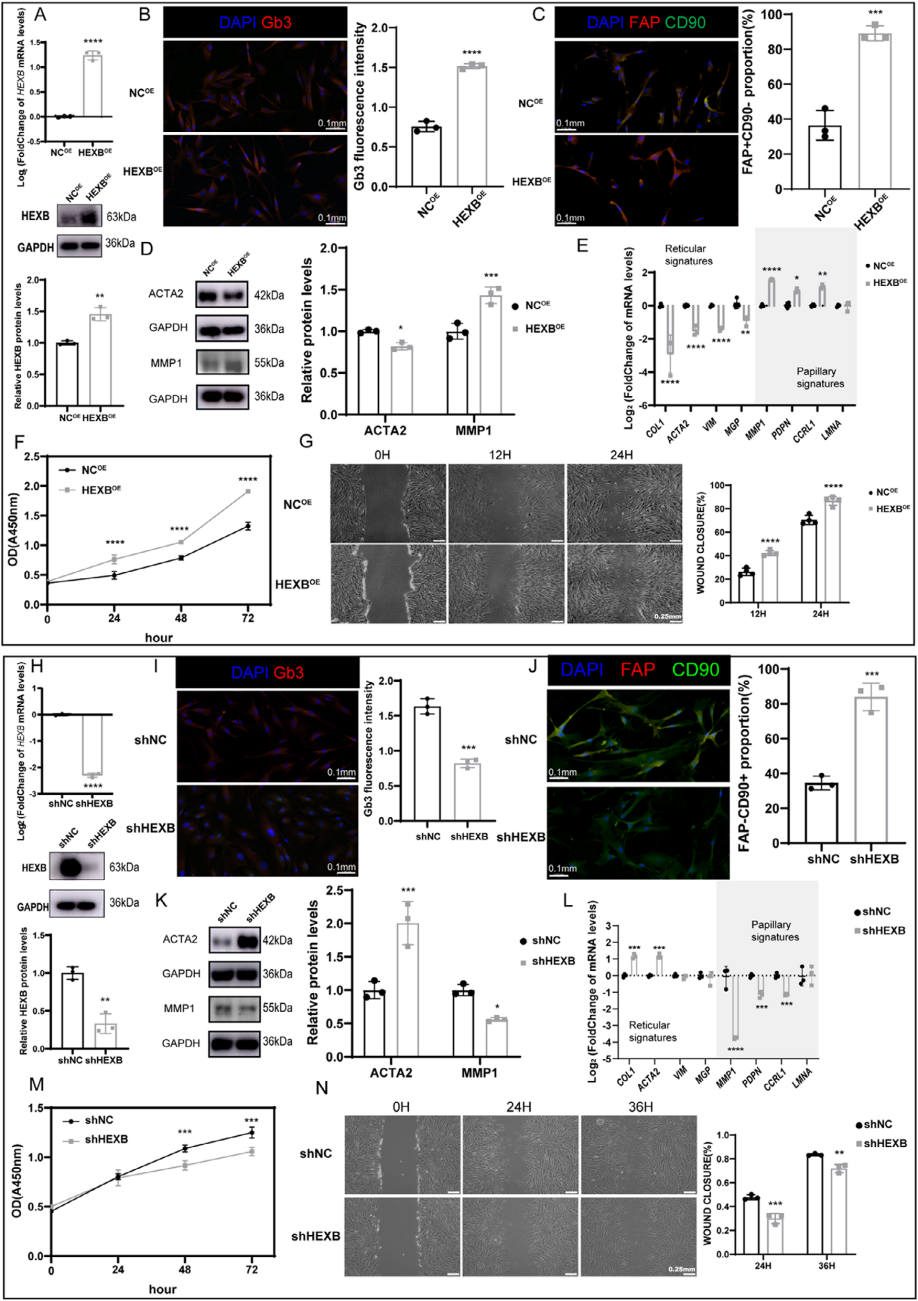
Alteration of HEXB resulted in codirectional alterations of Gb3 in fibroblasts affecting cell heterogeneity. A) Verification of HEXB overexpression at the mRNA and protein level in dHFBs transfected with HEXB^OE^, as detected by qRT‐PCR and western blot (*n* = 3). B) Representative immunofluorescence staining of Gb3 (red) in HEXB^OE^ and NC^OE^. Quantitative analysis of Gb3 fluorescence intensity in the experiments was shown on the right (*n* = 3). C) Representative immunofluorescence staining of FAP (red) and CD90 (green) in HEXB^OE^ and NC^OE^ cells. Quantitative analysis of FAP+CD90‐ proportion in the experiments was shown on the right (*n* = 3). D) Western blot and quantification of HEXB^OE^ and NC^OE^ cells. Data were normalized against GAPDH (*n* = 3). E) Barplots of qRT‐PCR quantifying the mRNA levels of papillary fibroblast and reticular fibroblast related genes in HEXB^OE^ and NC^OE^ cells. Data were shown as log_2_Fold Change (FC) over NC^OE^ cells (*n* = 3). F) CCK‐8 proliferation assay was used to demonstrate the proliferation of HEXB^OE^ cells (*n* = 3). G) Scratch test showed the migration of HEXB^OE^ cells (*n* = 3). *p*‐values were differences compared to: NC^OE^. H) Verification of HEXB knock‐down at the mRNA and protein level in dHFBs transfected with shHEXB, as detected by qRT‐PCR and western blot (*n* = 3). I) Representative immunofluorescence staining of Gb3 (red) in shHEXB and shNC (scale, 100 µm). Quantitative analysis of Gb3 fluorescence intensity in the experiments was shown on the right (*n* = 3). J) Representative immunofluorescence staining of FAP (red) and CD90 (green) in shHEXB and shNC. Quantitative analysis of FAP‐CD90+ proportion in the experiments was shown on the right (*n* = 3). K) Western blot and quantification of shHEXB cell and shNC cell. Data were normalized against GAPDH (*n* = 3). L) Barplots of qRT‐PCR quantifying the mRNA levels of papillary fibroblast and reticular fibroblast related genes in shHEXB cells and shNC cells. Data were shown as log_2_FC over shNC cells (*n* = 3). M) CCK‐8 proliferation assay was used to demonstrate the proliferation of shHEXB cells (*n* = 3). N) Scratch test showed the migration of shHEXB cells (*n* = 3). *p*‐values were differences compared to: shNC. The data are presented as the means ± SDs. *p < 0.05, **p < 0.01, ***p < 0.001, ****p < 0.0001.

To elucidate the impact of Gb3 on cellular heterogeneity in primary dHFBs, a series of experiments involving the transfection of dHFBs with HEXB‐associated lentivirus or control lentivirus were conducted. The aimed to investigate the regulation of cellular heterogeneity in dHFBs by HEXB overexpression (HEXB^OE^). The extent of HEXB overexpression was validated through quantitative real‐time polymerase chain reaction (qRT‐PCR) and western blot analysis (**Figure** **4**A). Notably, immunofluorescence quantification of Gb3 in dHFBs transfected with HEXB^OE^ or control lentivirus (NC^OE^) confirmed a significant increase in Gb3 levels in the overexpressed cells (Figure 4B). Subsequently, immunofluorescence staining on HEXB^OE^ cells and NC^OE^ cells was performed to observe the effects of Gb3 alteration on fibroblast heterogeneity. This revealed an augmentation in papillary fibroblasts characterized by FAP+CD90‐ (Figure 4C). Further analyses through western blot and qRT‐PCR demonstrated a notable increase in the expression of papillary fibroblast markers, such as MMP1 and a decrease in reticular fibroblast marker expression such as ACTA2 following HEXB overexpression in dHFBs (Figure 4D,E). Additionally, the proliferative and migratory abilities of dHFBs postintervention were evaluated through Cell Counting Kit‐8 (CCK‐8) proliferation assay and scratch assay, revealing enhanced proliferation and migration in HEXB^OE^ dHFBs, consistent with papillary fibroblast characteristics (Figure 4F,G).

The study then proceeded with loss‐of‐function approaches, involving transfection of dHFBs with lentivirus containing HEXB shRNA (shHEXB) or negative control shRNA (shNC) to generate shNC cells or HEXB‐knockdown shHEXB cells, respectively (Figure 4H). Quantitative immunofluorescence analysis revealed a marked attenuation of Gb3 immunoreactivity in shHEXB cells, demonstrating coordinated modulation of HEXB and its metabolic product (Figure 4I). Consequently, fibroblast heterogeneity was altered, evidenced by an increase in the number of reticular fibroblasts characterized by FAP‐CD90+ in shHEXB cells (Figure 4J). At the protein and RNA levels, a decrease in the expression of papillary fibroblast markers (e.g., MMP1) and an increase in reticular fibroblast markers expression (e.g., ACTA2) were observed following HEXB knockdown (Figure 4K,L). Functional experiments further corroborated these findings, indicating reduced cell proliferation and migration ability in shHEXB cells compared to shNC cells, aligning with the characteristics of reticular fibroblasts (Figure 4M,N). Collectively, these data establish HEXB as the principal enzymatic regulator of Gb3 biosynthesis, whose metabolic activity critically governs fibroblast lineage specification in SDB.

### 2.5 Gb3 Accelerates Wound Closure and Improves the Quality of Healing

**Figure 5:**
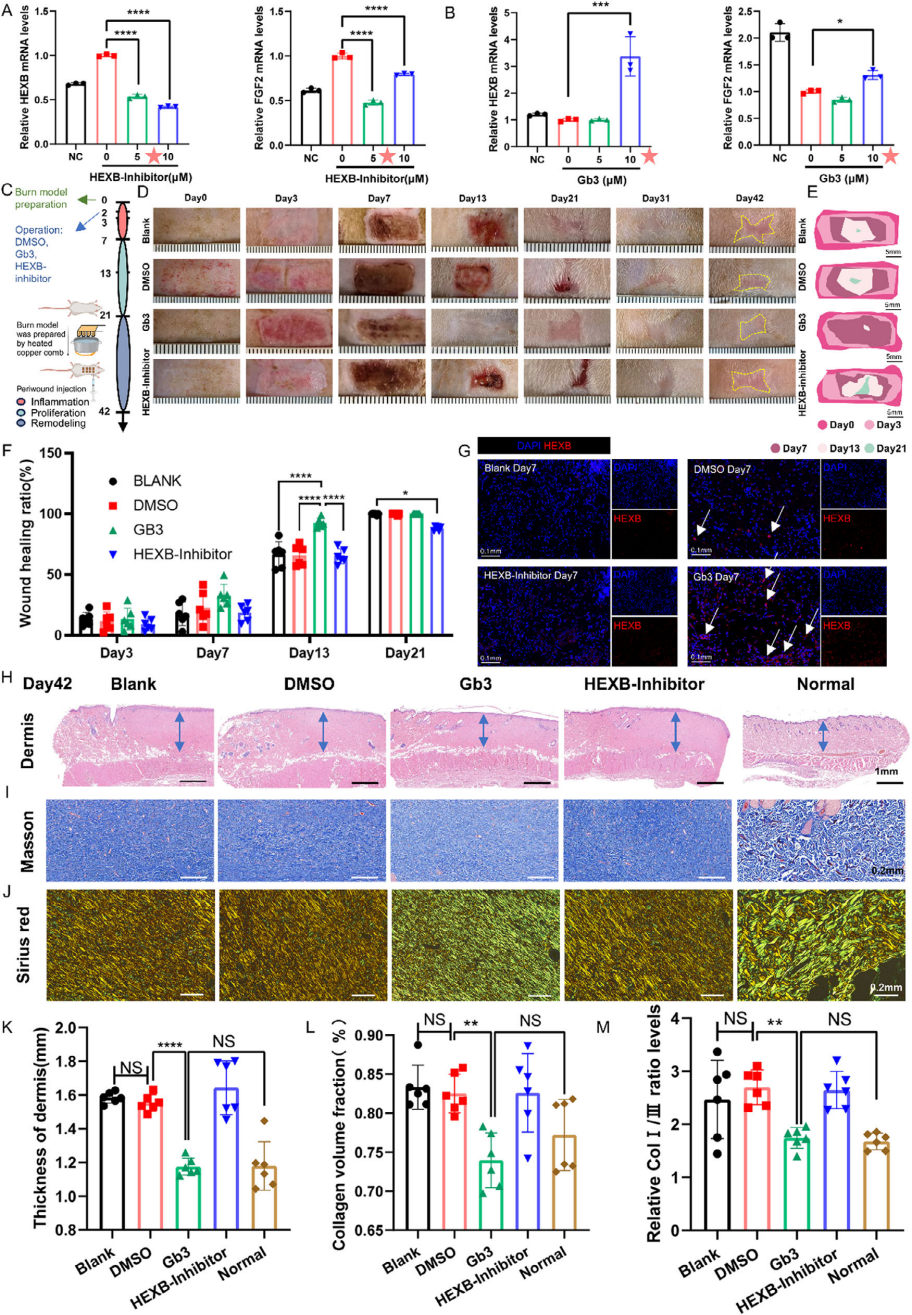
Evaluating the efficacy of Gb3 in promoting wound healing and preventing scar in vivo. A) Screening optimal drug treatment concentration with the qRT‐PCR. 48‐h cellular culture with 5 µm HEXB inhibitor was selected for subsequent experiments (*n* = 3). B) Screening optimal drug treatment concentration with the qRT‐PCR. 48‐h cellular culture with 10 µm Gb3 inhibitor was selected for subsequent experiments (*n* = 3). C) An experimental scheme of burn wound model. D) Visual representations of burn wounds. A yellow dashed line delineates the scar area. E) A diagram of appearance healing. F) Wound closure progression (*n* = 6). G) Immunofluorescence images demonstrate the effects of Gb3 and HEXB inhibitor on HEXB expression at the wound site. White arrows denote positive signals. H–J) Representative images of dermis thickness, Masson's trichrome staining, and Sirius red staining (Collagen I marked by red or orange; Collagen III marked by green) on day 42 postburn. K–M) Quantitation of dermis thickness, Masson's trichrome staining, and Sirius red staining among each group (*n* = 6). Statistical analysis was conducted using one‐way ANOVA. The data are presented as the means ± SDs. *p < 0.05, **p < 0.01, ***p < 0.001, ****p < 0.0001.

In order to investigate the impact of Gb3 on the healing process and scarring of burn wounds in living organisms, we established a burn model in Sprague–Dawley rats by applying a copper block heated to a temperature of 60 °C for a duration of 30 s.^[^ ^17^ ^]^ In animal experiments, we determined both the half‐maximal inhibitory concentration (IC_50_) and half‐maximal effective concentration (EC_50_) of the drug. The minimal effective concentration demonstrating low/high expression levels of downstream genes (HEXB and FGF2) was employed. The IC_50_ value of HEXB‐Inhibitor (6.81±0.45 µm) was relatively close to its EC_50_ value (3.40±0.29 µm). Considering both drug toxicity and pharmacological efficacy, a concentration of 5 µm was selected for subsequent experiments. In contrast, the IC_50_ of Gb3 (39.43±2.73 µm) was significantly higher than its EC_50_ (8.41±1.06 µm). Based on the dose‐response relationship, a concentration of 10 µm, which approximates the EC_50_ value, was chosen for further experimentation (**Figure** **5**A,B; and Figure S5, supporting Information). On the second day following the scorching of the rats' back skin, injections of different substances were administered: a solvent control group (DMSO), Gb3 group (10 µm), HEXB‐Inhibitor group (5 µm), and a blank control group (Blank). These injections were administered around the scaling site. Since DMSO is the officially recommended solvent for both Gb3 and HEXB‐Inhibitor reagents, we selected DMSO as the solvent control group. Considering the potential toxicity of DMSO, we injected 50 µL (0.18 g kg^−1^) per wound site. This dose is significantly lower than the previously reported median lethal dose (LD_50_) for DMSO (1 g kg^−1^ administered 5 days per week) in the literature.^[^ ^18^ ^]^

Wound tissues were harvested at multiple time points (Figure 5C). Based on the wound appearance, it was evident that Gb3 had a notable impact on facilitating the healing process of the burn wound. The Gb3‐treated group was the first to attain complete re‐epithelialization on day 13 postburn. However, the HEXB‐Inhibitor group, for which the HEXB enzyme related to Gb3 production was blocked, did not complete epithelialization on day 13 postburn. Both control groups (Blank and DMSO) completed re‐epithelialization within 21 days postburn (Figure 5D–F). The wounds in each group were assessed using the Vancouver Scar Scale (VSS, Table S2, supporting Information) on day 42 postburn, revealing that the Gb3 group exhibited the lowest VSS score, indicating the mildest scar severity (Figure S6A, supporting Information). Histological morphology at different time points across groups was demonstrated by hematoxylin and eosin (H&E) staining results (Figure S6B, supporting Information). Quantitative analysis confirmed enhanced healing kinetics in the Gb3‐treated group. Immunofluorescence results suggested that Gb3 and HEXB‐Inhibitors could effectively change the expression of HEXB on the wound surface (Figure 5G).

To determine the effect of Gb3 on complete wound repair, we included normal skin as the control group and analyzed dermal remodeling in each scald group on day 42. The results showed that the dermal thickness of the Gb3 group was similar to that of normal skin but thicker than the other groups (Figure 5H,K). Furthermore, using Masson's trichrome staining to assess dermal collagen deposition (Figure 5I,L), we found that the collagen volume in the Gb3 group was comparable to that of normal skin. These findings suggested that the Gb3 group increased granulation tissue remodeling, which in turn reduced the formation of scar tissue. We used Sirius Red staining to measure the ratio of type I to type III collagen (Col I/Col III) (Figure 5J,M). The results showed the ratio of the burn groups (Blank group, DMSO group, Gb3 group, and HEXB‐Inhibitor group) were greater than that of normal skin. This finding was consistent with the characteristics of hypertrophic scars following burns.^[^ ^19^ ^]^ Despite the fact that the Col I/Col III ratio of the Gb3 group was somewhat higher than that of the normal skin group, there was no statistically significant difference. This suggested that the scar‐free healing efficiency of the Gb3 group was the highest. These findings provided further evidence that Gb3 can accelerate burn wound healing and promote efficient dermal regeneration and remodeling during the remodeling phase.

### 2.6 Gb3 Regulates Cell Heterogeneity in dHFBs through FGF2 Signaling Pathway

**Figure 6:**
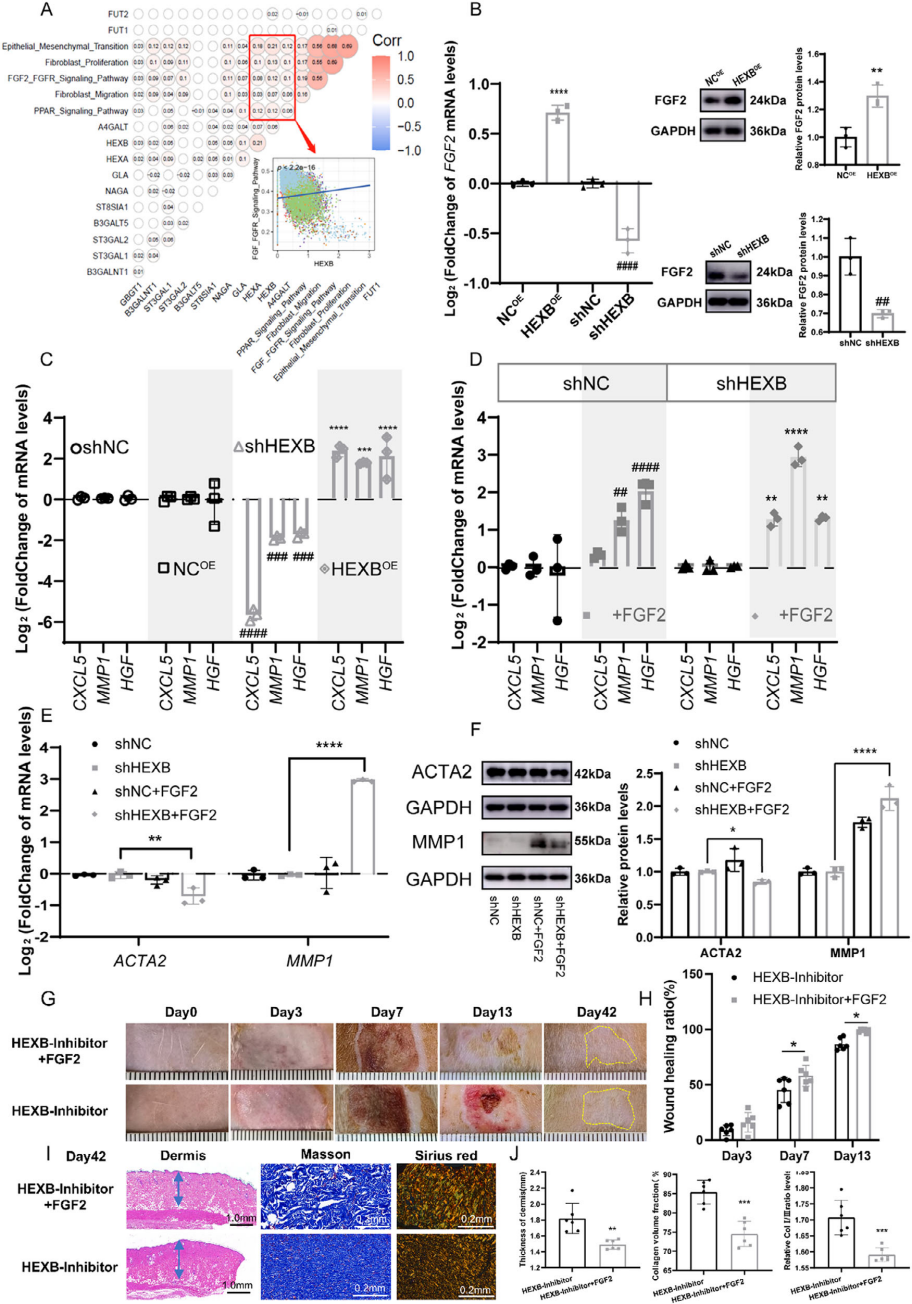
Correlation Analysis of Gb3 Synthesis and FGF2 Signaling Pathway. A) Correlation analysis scatter plots indicated that the FGF2‐FGFR signaling pathway was significantly activated in fibroblasts with active Gb biosynthesis, and the two were significantly positively correlated. B) Verification of FGF2 expression at the mRNA and protein level in dHFBs transfected with HEXB^OE^ and shHEXB, as detected by qRT‐PCR and western blot (*n* = 3). The data are presented as the means ± SDs. **p < 0.01, ****p < 0.0001 versus NC^OE^, ^##^ *p* < 0.01, ^####^ *p* < 0.0001 versus shNC. C) Barplots of qRT‐PCR quantifying the mRNA levels of FGF2 signal pathway genes in HEXB^OE^ cells and shHEXB cells. Data were shown as log_2_FC over untreated cells (*n* = 3). The data are presented as the means ± SDs. **p < 0.01, ****p < 0.0001 versus NC^OE^, ^##^ *p* < 0.01, ^####^ *p* < 0.0001 versus shNC. D) After 24 h of serum‐free starvation culture, shHEXB, and shNC cells were treated with FGF2 (MCE, HY‐P7330, 10 ng mL^−1^) for 24 h. The expression of FGF2 target gene mRNA was detected by qRT‐PCR. The data are presented as the means ± SDs. ***p < 0.01, ****p < 0.0001 versus shHEXB, ^###^ *p* < 0.01, ^####^ *p* < 0.0001 versus shNC. E,F) Western blot and quantitative real‐time PCR of cells treated as in (D). Data were normalized against GAPDH (*n* = 3). G) Visual representations of burn wounds. A yellow dashed line delineates the scar area. H) Wound closure progression. (*n* = 6) I) Representative images of dermis thickness, Masson's trichrome staining, and Sirius red staining (Collagen I marked by Red; Collagen III marked by green) on day 42. J) Quantitation of dermis thickness, Masson's trichrome staining, and Sirius red staining among each group *n* = 6. Statistical analysis was conducted using one‐way ANOVA. The data are presented as the means ± SDs. *p < 0.05, **p < 0.01, ***p < 0.001, ****p < 0.0001.

Next, we attempted to explore the mechanism by which Gb3 affects the heterogeneity of dHFBs. Previous studies have established that sphingolipid‐mediated regulation of fibroblast differentiation occurs via the FGF2 signaling pathway, with Gb3 acting as an activator and GM3 inhibiting the FGF2 function.^[^ ^9^, ^20^ ^]^ Therefore, it is intriguing to delve into the deeper and mutual regulatory mechanisms of the FGF2 signaling pathway, HEXB, and Gb3 synthesis in SDB fibroblasts. Initially, correlation analysis (**Figure** **6**A) revealed a positive correlation between the FGF2‐FGFR (Fibroblast Growth Factor Receptor) signaling pathway and HEXB expression, suggesting that the increase in Gb3 synthesis may activate this pathway, promoting fibroblast differentiation toward a papillary state, as described in a previous study.^[^ ^9^ ^]^

The expression levels of FGF2 in HEXB^OE^ and shHEXB cells were evaluated using western blot and qRT‐PCR techniques, revealing a consistent change in both FGF2 and HEXB levels (Figure 6B). Subsequent analysis of FGF2 downstream effectors revealed that the presence of Gb3 could influence their expression (Figure 6C). To explore whether the FGF2‐FGFR signaling pathway was regulated by Gb3, shHEXB cells and shNC cells were subjected to exogenous FGF2 factor intervention after 24‐h serum starvation. Remarkably, this intervention significantly increased the expression of downstream signaling molecules associated with FGF2 (Figure 6D). Moreover, administration of exogenous FGF2 factor intervention counteracted the upregulation of fibrotic markers induced by decreased Gb3 expression in reticular fibroblasts, inducing phenotypic switching to papillary fibroblasts with predominant fibrolytic marker expression (Figure 6E,F).

At the animal level, application of FGF2 after inhibiting Gb3 on burn wounds can enhance epithelialization and reduce scar formation. The rat burn wound model was created using the same method as previously described. On postburn day 2, HEXB‐Inhibitor was injected into the control group, and the experimental group received FGF2 (HEXB‐Inhibitor+FGF2). Wound topography showed that, compared with the control group, the HEXB‐Inhibitor+FGF2 group achieved complete epithelialization by day 13 (Figure 6G,H), with the wound site's skin color closer to normal skin by day 42 (Figure 6G). At the microlevel, dermal thickness, collagen volume, and Col I/Col III ratio of HEXB‐Inhibitor+FGF2 group were significantly lower than those of the control group (Figure 6I,J). Analysis of clinical samples by spatial enhanced resolution omics sequencing (Stereo‐seq) revealed significantly upregulated expression of the FGF2/FGFR signaling axis in SSDB compared with DSDB, suggesting its potential clinical relevance in burn wound healing (Figure S7, Supporting Information). These experiments demonstrated that Gb3 promotes a fibrolytic state in fibroblasts via the FGF2 signaling pathway and inhibits the myofibroblast state, thereby reducing burn scar formation.

### 2.7 Gb3 Modulates Cellular Heterogeneity in dHFBs through FGFR1 Binding

**Figure 7:**
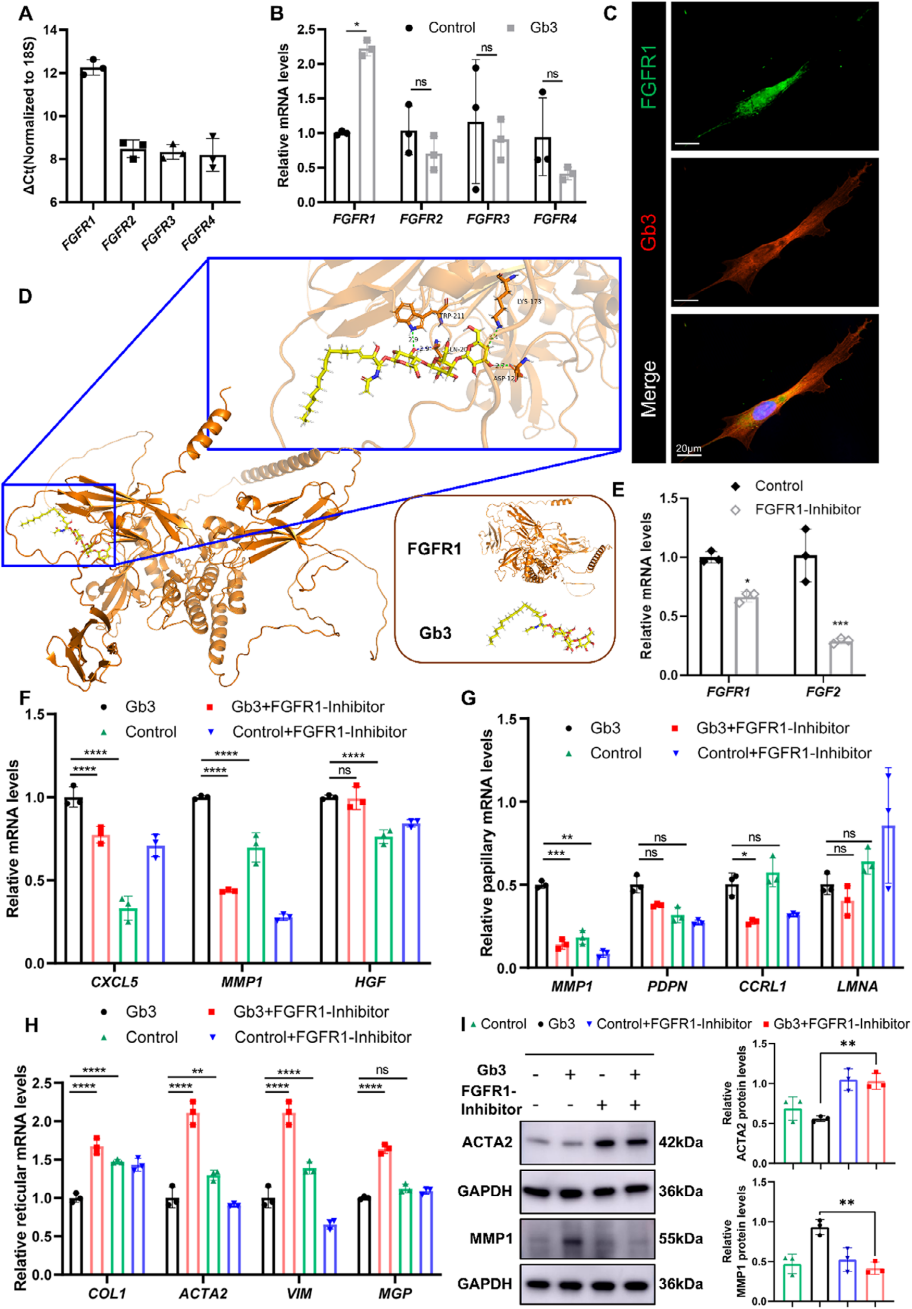
Recombinant FGF2 inhibits expression of pro‐fibrotic genes in dHFBs in an FGFR1‐dependent manner. A) mRNA from untreated early passage dHFBs was analyzed via qRT‐PCR for *FGFR1*, *FGFR2*, *FGFR3*, and *FGFR4*. ΔCt values were normalized to *18S*. B) After 24 h of serum‐free starvation culture, dHFBs were treated with Gb3 (10 µm) for 48 h. The expression of FGFR1 target gene mRNA was detected by qRT‐PCR. C) Representative immunofluorescence staining in dHFBs showing colocalization of Gb3 (red) and FGFR1 (green). D) Interaction between Gb3 and FGFR1. Residues of Gb3 interacting with FGFR1 were labeled and shown in blue stick model. Hydrogen bond is shown with green dash. E) After 24 h of serum‐free starvation culture, dHFBs were treated with FGFR1‐specific tyrosine kinase inhibitor PD173074 (MCE, HY‐P7330, 1 nm) for 48 h. The expression of FGFR1 target gene mRNA was detected by qRT‐PCR. F) Barplots of qRT‐PCR quantifying the mRNA levels of FGF2 signal pathway genes in control, Gb3, control+FGFR1‐inhibitor, and Gb3+FGFR1‐inhibitor cells (*n* = 3). G, H) Barplots of qRT‐PCR quantifying the mRNA levels of papillary fibroblast and reticular fibroblast related genes in control, Gb3, control+FGFR1‐inhibitor, and Gb3+FGFR1‐inhibitor cells (*n* = 3). I) Western blot of cells treated as in (F). Data were normalized against GAPDH (*n* = 3). The data are presented as the means ± SDs. *p < 0.05, **p < 0.01, ***p < 0.001, ****p < 0.0001.

Our preliminary findings suggest that Gb3 modulates the heterogeneity of dHFBs via the FGF2 signaling pathway. Consistent with established mechanisms, FGF2 signaling requires FGFR activation for downstream transduction. To determine which FGFR subtype is required for FGF2‐mediated MMP1 upregulation and ACTA2 suppression, we first quantified the relative expression of FGFRs in dHFBs using qRT‐PCR (**Figure** **7**A). Quantitative analysis identified FGFR1 as the predominant receptor subtype in dHFBs, with Gb3 treatment specifically modulating FGFR1 expression levels with statistical significance (Figure 7B). Subsequent immunofluorescence colocalization assays in dHFBs revealed an indirect interaction between Gb3 and FGFR1 (Figure 7C). Molecular docking simulations identified robust interactions between Gb3 and FGFR1, with hydrogen bonds formed between Gb3 and residues Gln209, Asp129, Trp211, and Lys173 (Figure 7D; and Figure S8, Table S3, supporting Information).

To investigate whether FGFR1 blockade modulates Gb3‐mediated fibroblast heterogeneity, we first validated the efficacy of FGFR1 inhibition. Administration of the FGFR1 inhibitor demonstrated a significant reduction in both FGF2 and FGFR1 expression levels (Figure 7E). Subsequent examination of FGF2 downstream signaling revealed that cotreatment with the FGFR1‐Inhibitor reversed the Gb3‐induced upregulation of downstream effectors in dHFBs. As shown in Figure 7F, comparative analysis between the Gb3‐treated group (black) and the control group (green) confirmed elevated expression of FGF2 downstream targets in the Gb3 group. Notably, the Gb3+FGFR1‐Inhibitor group (red) exhibited significant suppression of these downstream signaling components compared to the Gb3 monotherapy group (black).

Subsequently, we examined markers associated with papillary fibroblasts and reticular fibroblasts. In Gb3‐treated dHFBs cotreated with the FGFR1‐Inhibitor, we observed antagonistic effects between the inhibitor and Gb3 on fibroblast heterogeneity. Gb3 promoted fibroblast differentiation toward the papillary phenotype, while FGFR1‐Inhibition attenuated papillary differentiation and concurrently upregulated reticular fibroblast‐specific markers (Figure 7G,H). Furthermore, functional characterization of dHFBs revealed that FGFR1‐Inhibition reversed the Gb3‐induced upregulation of fibroblast remodeling‐associated markers, thereby promoting a transition toward a fibrogenic‐dominant reticular phenotype marked by increased expression of profibrotic genes (Figure 7I).

In conclusion, we synthesized the existing results and validated the essential scientific hypothesis that differences in Gb biosynthesis contribute to the heterogeneity of fibroblasts in DSDB and SSDB. Specifically, in SSDB, the upregulation of HEXB promoted Gb biosynthesis and sequentially activated the FGF2 signaling pathway, thereby enhancing fibroblast proliferative and fibrolytic abilities and leading to scar‐free healing. In DSDB, however, this pathway was suppressed, resulting in hypertrophic scar formation.

## 3 Discussion and Conclusion

Burn injury causes disfigurement, disability, and even mortality in patients, which leaves an enormous financial and mental burden on burn patients and their family members.^[^ ^21^ ^]^ Although patients are handled with optimal procedures, they often develop a hypertrophic scar on the healing wound,^[^ ^22^ ^]^ leading to disfigurement and dysfunction. The explicit mechanism of scar formation remains to be explored. In our study, we used Stereo‐seq to evaluate normal skin, DSDB, and SSDB wound clinical samples of our burn patients, and found differential Gb synthesis between DSDB and SSDB. Subsequently, spatial metabolomics integrated with scRNA‐seq analysis revealed that GbRGs, including *HEXB*, *HEXA*, *ST3GAL2*, and *A4GALT*, were predominantly expressed in proliferative and fibrolytic fibroblast phenotypes rather than reticular fibroblasts. Based on data from normal skin and scRNA‐seq results of burn wound skin, we identified *HEXB* as a key gene in burn wounds. Inhibition of HEXB through lentivirus has actuated several myofibroblast or collagen secretion markers’ perturbations; however, over‐expression of HEXB is more associated with papillary states. Considering these findings, HEXB may play a crucial role in mediating fibroblast properties and phenotype transition in DSDB and SSDB, potentially becoming a crucial treatment target for burn injury. Our research indicated a strong positive correlation between the FGF2 signaling pathway and HEXB, with notably higher expression in proliferative fibroblast phenotypes. The addition of exogenous FGF2 rescued the changes in fibroblast heterogeneity caused by HEXB knockdown. This step is mediated by the binding of Gb3 and FGFR1.

GSLs’ variation could influence cell differentiation and heterogeneity. For instance, along with the progression of embryonic stem cells in vitro differentiation, Gb4 was detected to switch to GM.^[^ ^23^ ^]^ GM synthesis also played an indispensable role in neural differentiation, whereas Gb repressed the expression of GM3 synthesis‐related genes, leading to the maldevelopment of the nervous system and neurocutaneous disorders like the Salt and Pepper syndrome.^[^ ^24^ ^]^ Likewise, a GM3 increment and ceramide dihexoside (CDH) reduction were observed in the transformation from human promyelocytic leukemia cell line HL‐60 to macrophage‐like cells in vitro, while the opposite GSL profiles were shown in the differentiation into myeloid mature cells.^[^ ^25^ ^]^ Our study identified differences in Gb3 between SSDB and DSDB and elucidated its downstream HEXB‐FGF2 signaling pathway, which held promise for innovative therapy for burn scars. The synthesis and degradation of GSLs involve a complex metabolic network that includes a variety of enzymes. Both A4GALT and HEXB are enzymes involved in Gb synthesis, each with distinct functions. LacCer is the metabolic branch point for the formation of the different classes of complex GSLs. For Gb3 (Galα1‐4Galβ1‐4Glcβ1‐Cer), the A4GALT (the α1‐4‐galactosyltransferase) plays an important role in the process by adding Gal to Galβ1‐4Glcβ1Cer (lactosylceramide, LacCer).^[^ ^13^ ^]^ HEXB can break down Gb4 (GalNAcβ(1‐3)Galα(1‐4)βGal(1‐4)βGlc‐Cer) to form Gb3, and is a key enzyme in another pathway of Gb3 synthesis.^[^ ^26^ ^]^ The abnormality of HEXB often causes pathological changes, such as Sandhoff Disease. Accordingly, our study identified significant differences in HEXB expression between SSDB and DSDB in burn wound skin, initially demonstrated in cell and animal experiments. This variation may result from burn‐induced stress triggering different signaling pathways compared to physiological conditions. Similar to the physiological state following food intake, carbohydrates are primarily absorbed as glucose in the small intestine and subsequently metabolized through aerobic oxidation to generate energy.^[^ ^27^ ^]^ However, during periods of stress, such as prolonged starvation, glucose is predominantly produced from lactate and glycerol. In this study, we demonstrated that the overexpression of HEXB, an enzyme related to Gb3 synthesis, contributed to the proliferation of papillary rather than reticular fibroblasts, thus preventing its further differentiation into myofibroblasts, excessive collagen secretion, and eventual scar formation. Therefore, it was speculated that Gb3 may be used to inhibit scar formation post burn, which would be the focus of our future study. Many enzymes involved in glycosphingolipid metabolism have become targets for therapeutic interventions. Enzyme replacement therapy (ERT) is based on chronic 2‐weekly infusion of (now recombinant) glucocerebrosidase targeted to macrophages by the presence of terminal mannose residues in its N‐linked glycans.^[^ ^28^ ^]^ Thus, given that HEXB is involved in Gb synthesis, it may serve as a targeted therapeutic agent for the treatment of burn wounds.

This study still has several limitations. First, because of ethical sampling requirements, the age and gender of patients represent confounding factors that may bias metabolomics analysis. We did not conduct a more extensive metabolic study of Gb3; however, we plan to intervene further in Gb3 within scars to investigate its impact on fibroblast heterogeneity and scar formation. Despite these limitations, our study is the first in the field of burn injuries to investigate whether differences in GSL metabolism between DSDB and SSDB affect dermal fibroblast phenotypes. Additionally, we established an intrinsic regulatory axis between GSL metabolism and fibroblasts, representing a significant innovation. As a comprehensive study involving bioinformatics analysis, cells, and animal experiments, further animal studies and clinical trials are warranted to validate our results and demonstrate the clinical relevance of the identified regulatory axis in burn wound fibroblasts. Furthermore, the clinical translation of GSL targeted therapies for burn wounds faces significant drug delivery challenges, primarily due to topical penetration barriers wherein burn eschar and dense fibrotic tissue impede drug penetration into deep dermal layers. While this study employs perilesional injection to ensure adequate drug delivery, noninvasive administration strategies are being prioritized for clinical translation. Future studies will investigate topical vesicular carriers or hydrogel‐based delivery systems to advance therapeutic applicability.

In conclusion, through the integration of bioinformatic analysis with cell and animal experiments, we uncovered a link between GSL metabolism and fibroblast phenotype changes in SDBs. Diversity in GSL biosynthesis, particularly Gb3, results in fibroblast heterogeneity between DSDB and SSDB. In SSDB, there is an increase in the expression of the Gb3‐related gene *HEXB*. Subsequently, HEXB promotes the synthesis of Gb3, and then activates the FGF2 signaling pathway through FGFR1, enhancing the proliferation and fibrolytic ability of fibroblasts, and ultimately promoting the differentiation of fibroblasts into the papillary subtype.

## 4 Experimental Section

### Data Collection

This study was approved by the Ethics Committee of the Shanghai Changhai Hospital (Approval NO. CHEC2023‐275). All procedures performed in studies involving human participants were in accordance with the ethical standards of the institutional and/or national research committee and with the 1964 Helsinki declaration and its later amendments or comparable ethical standards. The original scRNA‐seq of 9 DSDB samples, 3 SSDB samples from 7 to 26 days postburn, and 1 normal skin sample, together with 4 PMBC samples, were obtained from the burn patients treated at the Department of Burn Surgery in the First Affiliated Hospital of Naval Medical University (also named as Shanghai Changhai Hospital). Furthermore, 7 SSDB samples and 7 DSDB samples for Spatial Transcriptomics sequencing (Table S4, Supporting Information) were used. All clinical samples were taken from surgically discarded skin, and with the informed consent of the patients. 85 metabolic pathways and 1667 metabolic genes were achieved from the Kyoto Encyclopedia of Genes and Genomes (KEGG) database (https://www.kegg.jp/).^[^ ^29^ ^]^ The graphic flow diagram as well as the scientific hypothesis picture, were generated using Biorender (https://biorender.com/).

### Spatial Transcriptomics Analysis

Spatial transcriptomics (ST) slides were captured from 2 SDB patients for the investigation of gene expression profiles. The overall procedure was conducted by the Visium Spatial platform of 10x Genomics (https://www.10xgenomics.com/platforms/visium). Using spatially barcoded mRNA‐binding oligonucleotides, RNAs from the fixed and permeabilized tissue were released and bound to the barcodes on the Visium slide, thereby capturing the gene expression information. Then, cDNAs were synthesized from the above captured RNA. After mapping the raw sequencing data, the gene‐spot matrices underwent QC, normalization, and dimensional reduction across spots by the Seurat package (version 3.2.2, https://satijalab.org/seurat/).^[^ ^30^ ^]^ Spatial feature density plots were created with the Spatial Feature Plot function in Seurat.

### Lipidomic Analysis

Lipids were extracted from ≈30 mg of frozen tissues using a modified version of the Bligh and Dyer's method as described previously.^[^ ^31^ ^]^ Briefly, tissues were homogenized in 900 µL of chloroform: methanol: MilliQ H_2_O (3:6:1) (v/v/v). The homogenate was then incubated at 1500 rpm for 30 min at 4 °C. At the end of the incubation, 350 µL of deionized water and 300 µL of chloroform were added to induce phase separation. The samples were then centrifuged and the lower organic phase containing lipids was extracted into a clean tube. Lipid extraction was repeated once by adding 500 µL of chloroform to the remaining aqueous phase, and the lipid extracts were pooled into a single tube and dried in the SpeedVac under OH mode. Samples were stored at ‐80 °C until further analysis. Lipidomic analysis were conducted at LipidALL Technologies using a Shimadzu Nexera 20‐AD HPLC coupled with Sciex QTRAP 6500 PLUS as reported previously.^[^ ^32^ ^]^ Separation of individual lipid classes of polar lipids by normal phase (NP)‐HPLC was carried out using a TUP‐HB silica column (i.d. 150×2.1 mm, 3 µm) with the following conditions: mobile phase A (chloroform: methanol: ammonium hydroxide, 89.5:10:0.5) and mobile phase B (chloroform:methanol: ammonium hydroxide: water, 55:39:0.5:5.5). MRM transitions were set up for comparative analysis of various polar lipids.

### Processing of scRNA‐seq Data

The preprocessing of the scRNA‐seq data was carried out following the pipeline of the Singleron Matrix NEO (https://singleron.bio/services/single‐cell‐nucleus‐service/), and clean reads were then quantified by the Cell Ranger software (version 3.0, http://10xgenomics.com/). Subsequently, the quantified gene expression matrices were managed using the Seurat R toolkit (version 3.2.2, https://satijalab.org/seurat/).^[^ ^30^ ^]^ The doublets were identified and removed using the DoubletFinder R package.^[^ ^33^ ^]^ Transcript expression and the proportion of mitochondrial genes were considered indicators of QC, while only cells with less than 100 000 transcripts and less than 20% mitochondrial genes were included in the further analysis, and genes expressed in more than 15 single cells were included. After the strict QC process, the data were normalized, and the top 2000 highly variable genes were identified through “FindVariableFeatures” function. Stepwise, using the “ScaleData” function, the data were further transformed into a normal distribution. In order to reduce dimensionality, PCA was conducted, and the first 20 PCs were merged as input for Uniform Manifold Approximation and Projection for Dimension Reduction (UMAP) analysis for visualization.^[^ ^34^ ^]^ Cell cycle analysis was implemented by one of Seurat's functions, CellCycleScoring, which scored the cells according to their cell cycle phase, on the basis of previously defined genes involved in the cell cycle.^[^ ^35^ ^]^

### Cell Type Annotation

To recognize the cell type of each cluster, the “FindAllMarkers” function of Seurat was applied. Genes with an absolute value of log_2_FC higher than 0.50 (Wilcoxon rank‐sum test) as well as an adjusted *p*‐value < 0.05 (Bonferroni correction) in the top 2000 variable sets were considered to be DEGs and were utilized as a reference for cell type annotation.^[^ ^36^ ^]^ In addition, by comparing the guided marker genes above with the empirical cell markers from the CellMarker database, different unsupervised cell subsets were annotated and labeled.^[^ ^37^ ^]^ Cellular feature plots, violin plots, and heat maps were generated to show the marker genes of each cluster using SCANPY (Version: 1.7.1) and the Seurat R package (Version: 3.2.2) with Python 3.6.^[^ ^30^, ^38^ ^]^

### Cell–Cell Communication Analysis

Identifying and illustrating alterations in the intercellular signaling network (iTALK, https://github.com/Coolgenome/iTALK) was employed to recognize the cell–cell communication among different cell clusters.^[^ ^39^ ^]^ As one of the most important ways to transmit signals between any two cells, ligand–receptor binding indicates the crosstalk of particular cells and might help to build a communication network. Based on the top 500 highly expressed genes in all cells, this R package detected the top 80 most relevant ligand–receptor pairs (LRPs), and they were delineated by LR plots and iTALK networks. The iTALK analysis was beneficial to parse the complicated intercellular signaling networks among the cell clusters have defined.

### Quantification and Differential Expression Analysis of Metabolic Activity

85 metabolic pathways from the KEGG database were brought into the study. With the application of the “scMetabolism” function,^[^ ^40^ ^]^ the activity of these metabolic pathways was scored according to the genes and their average expression in the particular pathway. Afterward, metabolic pathways with significantly different activities between DSDB and SSDB, SSDB and normal tissue, and DSDB and normal tissue were plotted as heat maps, respectively (*p*‐value < 0.05), and the 3 metabolic pathway sets above were intersected to select the 49 significant pathways among the 3 types of tissues.

### Gene Set Variation Analysis (GSVA)

With the scored metabolic pathways above, GSVA was performed by GSVA package (Version: 1.38.0)^[^ ^41^ ^]^ to calculate the definite quantification for different 49 metabolic pathway activities in terms of the grade of SDB and the number of days postburn. The corresponding integrated or independent violin plots were created. GSVA was also utilized in ST analysis to identify the active density of metabolic pathways at the spatial level in SDB tissues.

### Spatial Enhanced Resolution Omics‐Sequencing (Stereo‐seq)

With high sensitivity, a large field of view and single cell resolution, Stereo‐seq technology was conducted to generate spatial transcriptomic data from human skin SDB tissue. The mRNAs in tissue were captured by Stereo‐seq chip and restored to their spatial position via coordinate identity (CID), realizing the spatial information detection.^[^ ^12^ ^]^ The Stereo‐seq chip was covered with DNA nanoball (DNB) arranged on the patterned arrays, which were generated by tolling circle amplification of single‐stranded circular DNA templates. Next, it was incubated with CID sequencing primer in MGI DNBSEQ‐Tx sequencer by single‐end sequencing, in order to determine particular DNB‐CID sequences at every spatial location. Then, capture probes were prepared by ligating unique molecular identifiers (UMI) and poly‐T including oligonucleotides to each locus. Followingly, snap‐frozen SDB skin tissue sections were adhered onto the chip surface, and then fixed and permeabilized to capture poly‐A‐tailed RNA, which were reversed transcribed as well as amplified. The resulting cDNA products were utilized as template for Stereo‐seq library construction, and were sequenced along with CID. Ultimately, the raw sequencing data were processed under the guidance of SAW pipeline (https://doi.org/10.46471/gigabyte.111) for high‐resolution spatial resolved transcriptomic analysis.

### Spatial Metabolomics Integrated scRNA‐seq Analysis

The lipid identity data attained by matrix‐assisted laser desorption/ionization mass spectrometry imaging (MALDI‐MSI) from dHFBs were uploaded and annotated on the METASPACE platform (https://metaspace2020.eu/),^[^ ^42^ ^]^ according to a recent article with great innovation.^[^ ^9^ ^]^ The metabolites at different points in a slice were extracted, and the abundance of each was measured by MS technique, before showing the distribution of them on the images. In its public data, the scRNA‐seq data of dHFBs samples with distinct treatments were extracted, which were 1) dHFBs with sphingolipid production inhibitors, fumonisin B1 (FB1), 2) dHFBs with overexpressing glycosphingolipid globo‐series 4 synthetase (OEGb4S), 3) dHFBs with overexpressing glycosphingolipid ganglio‐series 3 synthetase (OEGM3S), 4) dHFBs with an empty vector or transgene (EVT), and 5) control group. The previously described scRNA‐seq data processing procedures were then repeated to identify 6 dHFBs subtypes in 9 samples.

### Differential Expression Analysis Based on the Grade of Second‐Degree Burn

The DEGs between DSDB and SSDB, SSDB and normal tissue, and DSDB and normal tissue were identified using an edgeR R package,^[^ ^43^ ^]^ with an absolute value of log_2_ FC greater than 0 and an adjusted *p*‐value less than 0.05 considered the cutoff standard. Following that, the DEGs of SSDB and DSDB were intersected with Gb synthesis‐related metabolic genes from the KEGG database to identify the significant target genes in second‐degree burned skin and the Gb synthesis pathway. The visualization relied on the Venn diagram.^[^ ^44^ ^]^

### Isolation and Culture of Dermal Human Fibroblasts

As described previously,^[^ ^9^ ^]^ primary dermal human fibroblasts were prepared with the Naval Medical University from the dermis of discarded skin samples derived from the foreskins of circumcised 1 to 5‐year‐old healthy males, with patient and institutional approvals. Foreskin tissue samples were obtained following standard protocols and ethical guidelines. Briefly, adherent subcutaneous adipose tissue and deeper hypodermal layers were meticulously dissected and removed. The processed tissue samples were immersed in 1 U mL^−1^ Dispase II (Sigma‐Aldrich, NO. D4693) and incubated overnight at 4 °C. Following incubation, the epidermal layer was carefully discarded, and the remaining dermal tissue was collected for further processing. The isolated dermis was minced into small fragments (≈1–2 mm^3^) and subjected to enzymatic digestion in HBSS (without Ca^2^⁺ and Mg^2^⁺) containing 0.3% collagenase type I (Sigma‐Aldrich, NO. 1 148 089), using a ratio of 1 gram of tissue per 2 mL of enzyme solution. The tissue‐enzyme mixture was incubated at 37 °C for 1 h with gentle agitation. Digestion was terminated by adding an equal volume of DMEM supplemented with 10% v/v fetal bovine serum (FBS). The resulting cell suspension was sequentially filtered through a 40‐µm cell strainer to remove undigested tissue debris. Cells were pelleted by centrifugation at 2000 rpm for 5 min. The cell pellet was resuspended in complete growth medium, consisting of DMEM supplemented with 10% v/v FBS, 1 g L^−1^ glucose, 2 mm L‐glutamine, and 1 U mL^−1^ penicillin/streptomycin. Cells were plated at a density of 1×10⁵ cells per 10‐cm culture dish. Cultures were maintained in a humidified incubator at 37 °C under a controlled atmosphere of 5% CO_2_. After 48 h of initial plating, the culture medium was aspirated to remove nonadherent cells and debris, and fresh complete growth medium was added. By this stage, adherent cells exhibiting a characteristic spindle‐shaped morphology were readily observable (Figure S9A, Supporting Information).

### Cell Characterization and Quality Control

Cellular identity was confirmed by flow cytometry and immunofluorescence staining. Approximately 95% of the cultured cells exhibited high expression levels of the fibroblast markers Vimentin (VIM) and Collagen Type I (COL1) (Figure S9B–D, Supporting Information). Contamination by epithelial or endothelial cells was excluded based on negligible expression of Keratin 14 (KRT14) and CD31 (≈1%), respectively (Figure S9E, Supporting Information). To ensure cell line authenticity and absence of contamination, cells at early passages underwent mycoplasma detection testing and Short Tandem Repeat (STR) profiling. Results confirmed the absence of mycoplasma contamination and ruled out cross‐contamination with other cell lines (Files 1 and 2, Supporting Information).

### Cell Culture and Lentivirus Transfection

Cells were grown in DMEM supplemented with 10% v/v fetal bovine serum (FBS), 1 g L^−1^ glucose, 2 mm L‐glutamine, 1 U mL^−1^ enicillin/streptomycin and under a controlled atmosphere in the presence of 5% CO_2_ at 37 °C. Primary dermal human fibroblasts at passages 3–5 were used for lentivirus transfection. Lentivirus expressing a shRNA targeting HEXB and lentivirus selectively expressing HEXB, both of which were synthesized by Shanghai GeneChem Co., Ltd. (Shanghai, China), were added to dermal human fibroblasts to knockdown or overexpress HEXB, respectively. For the control, a lentiviral vector that expresses GFP alone was used. Then the transfected fibroblasts were cultured in DMEM with 10% fetal bovine serum. After transfection, 2.0 µg mL^−1^ puromycin was used to screen stable cell lines. The expression of HEXB mRNA and protein were detected to determine whether the overexpression or knockdown of HEXB was successful.

### Flow Cytometry

Cells (1 × 10⁶ per marker) were fixed and permeabilized using the Cyto‐Fast Fix/Perm Buffer Set (BioLegend, #426 803) following manufacturer's instructions. For intracellular targets [VIM (CST, NO.D21H3, 1:100), COL1 (abcam, NO.ab138492, 1:100), KRT14 (abcam, NO.ab119695, 1:100)], staining was performed postpermeabilization. Surface marker CD31 (abcam, NO.ab24590, 1:100) was stained prior to fixation. After primary antibody incubation (30 min, RT, dark), cells were washed and incubated with species‐matched Alexa Fluor 594 secondary antibodies: Goat anti‐Mouse (Invitrogen, NO.A‐11032, 1:100) for VIM, COL1 and KRT14, or Goat anti‐Rabbit (Invitrogen, NO.A‐11012, 1:100) for CD31 (30 min, RT, dark). Washed cells were resuspended in PBS and analyzed using a Flow cytometer (CytoFLEX) with appropriate controls. All the RRID information of the antibodies used in this section is placed in Table S5 (Supporting Information).

### Western Blot

Proteins from cell samples were lysed by RIPA buffer at 4 °C for 30 min, followed by centrifugation. The supernatant was harvested to quantify the protein concentration using a bicinchoninic acid assay (BCA) kit. After sodium dodecyl sulfate poly‐acrylamide gel electrophoresis (SDS‐PAGE), the protein was transferred to PolyVinylideneFluoride (PVDF) membranes and incubated overnight at 4 °C with primary antibodies (HEXB, Santa Cruz, NO.sc‐376781, 1:1000; ACTA2, Abcam, NO.ab7817, 1:1000; MMP1, Cell Signaling Technology, NO.E9S9N, 1:1000; FGF2, Santa Cruz, NO.sc‐74412, 1:1000; GAPDH, Epizyme Biotech, NO.LF206, 1:1000), followed by secondary antibodies anti‐Mouse IgG‐HRP (Epizyme Biotech, NO.LF101, 1:2000) or anti‐Rabbit IgG‐HRP (Epizyme Biotech, NO.LF102, 1:2000). ImageJ software was implemented to calculate the protein expression of the collected bands. All the RRID information of the antibodies used in this section is placed in Table S5 (Supporting Information).

### Quantitative RealTime ‐ PCR (qRT‐PCR)

A TRIzol reagent was performed to extract the total RNA from fibroblasts, the concentration of which was quantified through a NanoDrop (Thermo Fisher Scientific). qRT‐PCR was applied using SYBR Green PCR Master Mix, and 18S served as the internal control. PCR primers are listed in Table S6 (Supporting Information).

### Cell Proliferation Assay

The proliferation of fibroblasts was assessed using Cell Counting Kit‐8 (CCK‐8). Cells were seeded and cultured in 96‐well plates with 100 µL 10% CCK‐8 reagent for 2 h at 37 °C. The absorbance was measured at 450 nm. All experiments were conducted in triplicate.

### Cell Scratch Assay

Fibroblasts were seeded in a 6‐well plate. As the cells reached 100% confluence, they were scratched by a 200‐µL pipette tip. In order to eliminate the proliferation effect, the antimitotic mitomycin C was added to the plate.^[^ ^45^ ^]^ The images were attained by an Olympus light microscope.

### Immunofluorescence

Cells in 6‐well chambers were fixed in PBS containing 4% formaldehyde (10 min) and blocked with 5% BSA in PBS containing (8 h). Between each step, the samples were washed 3 times with PBS. Normal skin, SSDB, and DSDB wound skin tissue samples came from patients undergoing surgery. The discarded wound skin tissue of burn patients was taken to prepare frozen sections. After rehydration, the frozen sections were permeabilized (treated with 0.2% Triton X‐100‐H_2_O at room temperature for 30 min) and then the samples were blocked by 5% BSA in PBS containing (8 h). To stain different proteins, cells were incubated overnight at 4 °C with primary antibodies (HEXB, Santa Cruz, NO.sc‐376781, 1:100, Proteintech, NO.16229‐1‐AP. 1:100; FAP, Abclone, NO.A23789, 1:100; CD90, Santa Cruz, NO.sc‐53456, 1:100; Gb3, GeneTex, NO.GTX30743, 1:200; FGFR1, CST, NO.D8E4, 1:200; VIM, CST, NO.D21H3, 1:200, COL1, abcam, NO.ab138492, 1:200, KRT14, abcam, NO.ab119695, 1:200, CD31, abcam, NO.ab24590, 1:200). The slides were washed 3 times in PBS for 5 min. Secondary antibodies, goat antirabbit Alexa488 (Invitrogen, NO. A11008, 1:300), goat antimouse Alexa594 (Invitrogen, NO. A11005, 1:300) and goat antirat Alexa488 (Invitrogen, NO. A48262TR, 1:300) were added and incubated for 2 h at room temperature in the dark. The slides were then washed 3 times with PBS for 5 min. Nuclei were stained with DAPI (1:100 in PBS, 10 min). Imaging was performed on a Nikon‐Ti‐E inverted fluorescence microscope to scan the picture. All the RRID information of the antibodies used in this section is placed in Table S5 (Supporting Information).

### In Vitro Cytotoxicity Assay and Dose‐Response Relationship Analysis

The in vitro cytotoxicity of the drug against dHFBs was assessed using the CCK‐8 assay. Based on literature^[^ ^46^ ^]^ and preliminary experimental results, the following concentration gradients were established: FGFR1‐Inhibitor (nm): 0, 1, 5, 10, 15, 20, 25, 50, 100; FGF2 (ng mL^−1^): 0, 1, 5, 10, 15, 25, 50, 100, 300; Gb3 (µm): 0, 1, 5, 10, 15, 20, 25, 50, 100; HEXB‐Inhibitor (µm): 0, 1, 2, 4, 8, 16, 32, 64, 128.

All data were analyzed using Prism 8 software (GraphPad) to determine the IC_50_ of each compound for dHFBs. Following preliminary cytotoxicity screening, dHFBs were treated according to these concentration gradients. After drug exposure, cells were harvested for qRT‐PCR analysis of key gene expression levels. Data processing was performed using Prism 8 software to calculate the EC_50_ of each compound in dHFBs. All assays were conducted in triplicate across three independent experiments. Measurement data are expressed as mean ± standard deviation (S.D.). Corresponding data are presented in Figures S5 and S10 (Supporting Information).

### Establishment of an Animal Burn Model

The animal experiments received approval from the IEC for Clinical Research and Animal Trials at the Ethics Committee of Shanghai Changhai Hospital (Approval NO. CHEC(A.E.)2023‐038). All applicable institutional and/or national guidelines for the care and use of animals were followed. For this study, the 8‐week‐old male Sprague‐Dawley rats from Gem Pharmatech Co., Ltd was procured. Following the established protocol, the 8‐week‐old male rats were sedated with isoflurane and positioned on the surgical table. Following immersion in hot water at 60 °C for 15 min, copper blocks were applied to the dorsal skin of depilated rats for 30 s to create burn models.^[^ ^17^ ^]^

Four square scalded lesions measuring 1 cm by 1.5 cm were created on the left and right sides of each rat's back. Utilize medical gauze for dressing to prevent wound infection and further harm. Medication of the wound is permissible on the second day postburn. Gb3 (globotriaosylceramide, Cayman Chemical, NO. CAY‐24870‐1), HEXB inhibitors (M‐31850, MCE, NO. HY‐104050) and FGF2 (MCE, NO. HY‐P7091) were solubilized in DMSO (MCE, NO. HY‐Y0320) to establish the requisite drug concentrations. The animal tests were categorized into four groups: 1) Control group without intervention (Control group); 2) Solvent control group injected with DMSO around the wound (DMSO group). 3) The Gb3 overexpression group received an injection of Gb3 during the trauma week (Gb3 group); 4) The Gb3 inhibitor group was administered an inhibitor of the Gb3 synthesis‐related enzyme HEXB (HEXB‐inhibitor group); 5) The FGF2 rescue group was administered HEXB‐inhibitor and recombinant rat FGF2 protein. Skin samples were taken on days 3, 7, 13, and 42 postburn and preserved in 4% paraformaldehyde for wax block preparation. Skin tissue wax blocks were sectioned to a thickness of 5 µm for further staining investigation.

### Wound Healing Analysis

Wounds were recorded and photographed at 0, 3, 7, 13, 21, 31, and 42 days post‐treatment. Wounds with intact dressings were maintained for study, whereas those with absent or detached dressings were excluded. The ImageJ software was utilized to investigate the wound region.

(1) $$\mathrm{Wound}\;\;\mathrm{healing}\;\;\mathrm{ratio}\;\left(\%\right)=\left(A_{0}-A_{t}\right)/A_{0}\times 100\%$$

where *A*_t_ represents the area at each time point, *A*_0_ represents the initial.

### Hematoxylin‐Eosin Staining

To dewax the sections, the prepared skin tissue samples were sequentially immersed in fresh xylene solution I for 15 min, xylene solution II for 15 min, xylene solution III for 15 min, anhydrous ethanol I for 6 min, anhydrous ethanol II for 6 min, anhydrous ethanol III for 6 min, 75% alcohol for 6 min, and ultimately rinsed with PBS. The sections were submerged in the hematoxylin dye (Servicebio, NO. G1005) solution for 5–6 min, followed by nuclear staining. The sections underwent dehydration using a gradient of 75%, 85%, and 95% alcohol for 5 min per cylinder; thereafter, the sections were immersed in eosin stain (Servicebio, NO. G1005) solution for 5–6 min, resulting in cytoplasmic staining. Ultimately, the skin sections were dyed following dehydration with ethanol and rendered transparent using xylene. The stained sections were ultimately sealed with neutral glue and examined under a microscope. Sinopharm Chemical Reagent Co., Ltd. provided other reagents in addition to hematoxylin and eosin dye.

### Masson's Trichrome Staining

The dewaxing procedure is outlined in the Hematoxylin‐Eosin Staining protocol. Submerge the dewaxed sections in Masson A solution for ≈16 h, then thoroughly rinse under running water. Immersed in Masson B solution and Masson C solution for 1 min, subsequently rinsed with running water, separated with 1% hydrochloric acid alcohol, and rinsed again with running water. Thereafter, Masson D solution, Masson E solution, and Masson F solution were immersed and dyed for 5 min, 1 min, and 5 s, respectively. The specimen was rinsed and differentiated with 1% glacial acetic acid, dehydrated with anhydrous ethanol, and turned transparent with xylene. The stained sections were subsequently sealed with neutral adhesive and analyzed microscopically.

### Sirius Red Staining

The hematoxylin‐eosin staining protocol detailed the dewaxing process. Iron hematoxylin staining solution was applied to the dewaxed sections for 5 min, then sections were rinsed with running water for 5 min. After the Sirius red stain (Servicebio, NO. GP1138) drops had been applied for 15 min, sections were rinsed with running water gently to eliminate the surface stain on the sections. Anhydrous ethanol was dehydrated, and xylene was translucent. The neutral gum seal was ultimately examined using a polarized light microscope. Sirius red‐stained slices were examined using Polarized light microscope (Nikon, Japan) to locate and quantify collagen deposition in the ECM surrounding both injured and normal skin.^[^ ^47^ ^]^

### Molecular Docking

The molecular docking study was performed between the target proteins and ligand molecules based on the structural files of Gb3 (PubChem CID: 66 616 222) and FGFR1 (UniProt ID: P11362). The Molecular Operating Environment (MOE) 2019 software suite was employed for protein preparation and molecular docking simulations, following established computational protocols.^[^ ^48^ ^]^ A total of 30 ligand conformations were generated through systematic conformational sampling using the default MOE algorithm. The resulting conformations were subsequently ranked according to their docking scores calculated by the London dG scoring function, with the top five highest‐scoring poses selected for further analysis. The optimal binding conformation was determined through a comprehensive evaluation of ligand–protein interaction patterns and binding site complementarity. Molecular visualization and interaction diagram generation were conducted using PyMOL Molecular Graphics System (version 2.5.2).^[^ ^49^ ^]^

### Quantitative Statistical Analysis

All statistical analysis processes were applied by R version 4.0.3 (Institute for Statistics and Mathematics, Vienna, Austria; www.r‐project.org), Strawberry Perl version 5.30.0.1 software (https://www.perl.org/), Python version 3.6 software (https://www.python.org/), and Prism 8 software. For descriptive statistics, mean ± standard deviation was utilized for continuous variables in a normal distribution. And for continuous variables in abnormal distribution, the mean (range) was used. Moreover, counts and percentages were used to describe the categorical variables. Statistical analyses included a two‐tailed unpaired Student's *t*‐test to compare the difference between paired groups and a one‐way analysis of variance (ANOVA) with a Bonferroni post‐test to assess the significance across the groups. Only a two‐sided *p*‐value < 0.05 and an FDR < 0.05 were considered statistically significant in this study.

## Conflict of Interest

The authors declare no conflict of interest.

## Author Contributions

S.X., R.H., W.Q., and X.D. contributed equally to this work. S.X., R.H., W.Q., X.D., and S.J. conceptualized and designed the project. S.X., R.H., and W.Q. performed scRNA‐seq analysis, spatial transcriptomics analysis, and lipidomic analysis. S.X., W.Z., X.D., Y.L., and J.L. performed in vivo studies. S.X., W.Z., H.S., Y.L., and B.L. performed in vitro experiments. S.X., R.H., W.Q., and X.R. conducted statistical analysis of the data. M.J. and Z.X. provided critical insights and advice. S.X., R.H., W.Q., and X.D. obtained the data and wrote the manuscript. M.W., Z.X., and S.J. supervised the study.

## Supporting information



Supporting Information

Supporting Information

## Data Availability

Generated data and codes can be acquired from the corresponding author on a reasonable request. The relevant code can be found in File3 (Supporting Information).
