# Supplementary material for: Globotriaosylceramide Gb3 Influences Wound Healing and Scar Formation by Orchestrating Fibroblast Heterogeneity
Source: Adv Sci (Weinh). 2025 Aug 14;12(41):e09733. doi: 10.1002/advs.202509733 (PMC12591104; doi:10.1002/advs.202509733)
Supplement: Supplementary file 1 — Supporting Information [file ADVS-12-e09733-s002.docx]

**Globotriaosylceramide Gb3 Influences Wound Healing and Scar Formation by Orchestrating Fibroblast Heterogeneity**

*Sujie Xie, Runzhi Huang*, Weijin Qian, Xinran Ding, Wei Zhang, Yixu Li, Jianyu Lu, Hanlin Sun, Yifan Liu, Yuntao Yao, Bingnan Lu, Minjuan Wu*, Zhaofan Xia*, Shizhao Ji**

**Affiliations:**

Sujie Xie, Runzhi Huang, Xinran Ding, Wei Zhang, Yixu Li, Jianyu Lu, Hanlin Sun, Zhaofan Xia, Shizhao Ji

Department of Burn Surgery, the First Affiliated Hospital of Naval Medical University, Shanghai 200433, China.

Email: shizhaoji@smmu.edu.cn (Shizhao Ji); [xiazhaofan_smmu@163.com](mailto:xiazhaofan_smmu@163.com) (Zhaofan Xia); runzhihuang@smmu.edu.cn (Runzhi Huang).

Weijin Qian

Department of Ophthalmology, Shanghai Ninth People's Hospital, Shanghai Jiao Tong University School of Medicine, Shanghai 200011, China.

Yifan Liu, Yuntao Yao, Bingnan Lu

Department of Urology, Xinhua Hospital Affiliated to Shanghai Jiao Tong University School of Medicine, Shanghai 200092, China.

Yifan Liu, Yuntao Yao

BGI research, BGI-Hangzhou, Hangzhou 310012, China.

Minjuan Wu

Department of Histology and Embryology, Naval Medical University, Shanghai 200433, China.

Email: minjuanwu2020@smmu.edu.cn.

Co-first authorship: Sujie Xie, Runzhi Huang, Weijin Qian, and Xinran Ding have contributed equally to this work.

# Figures


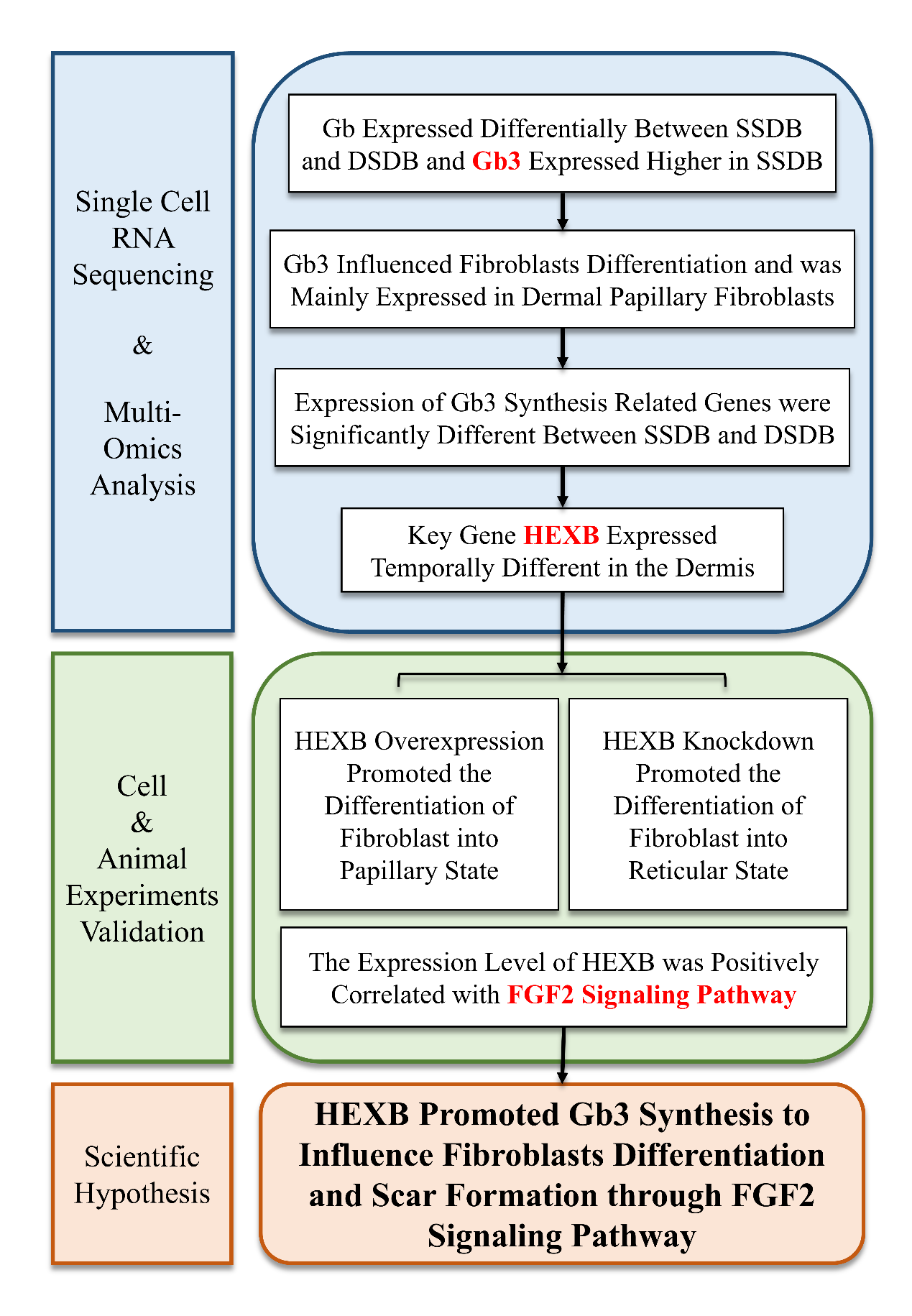


**Supplementary Figure 1 The working flow chart of this study.**


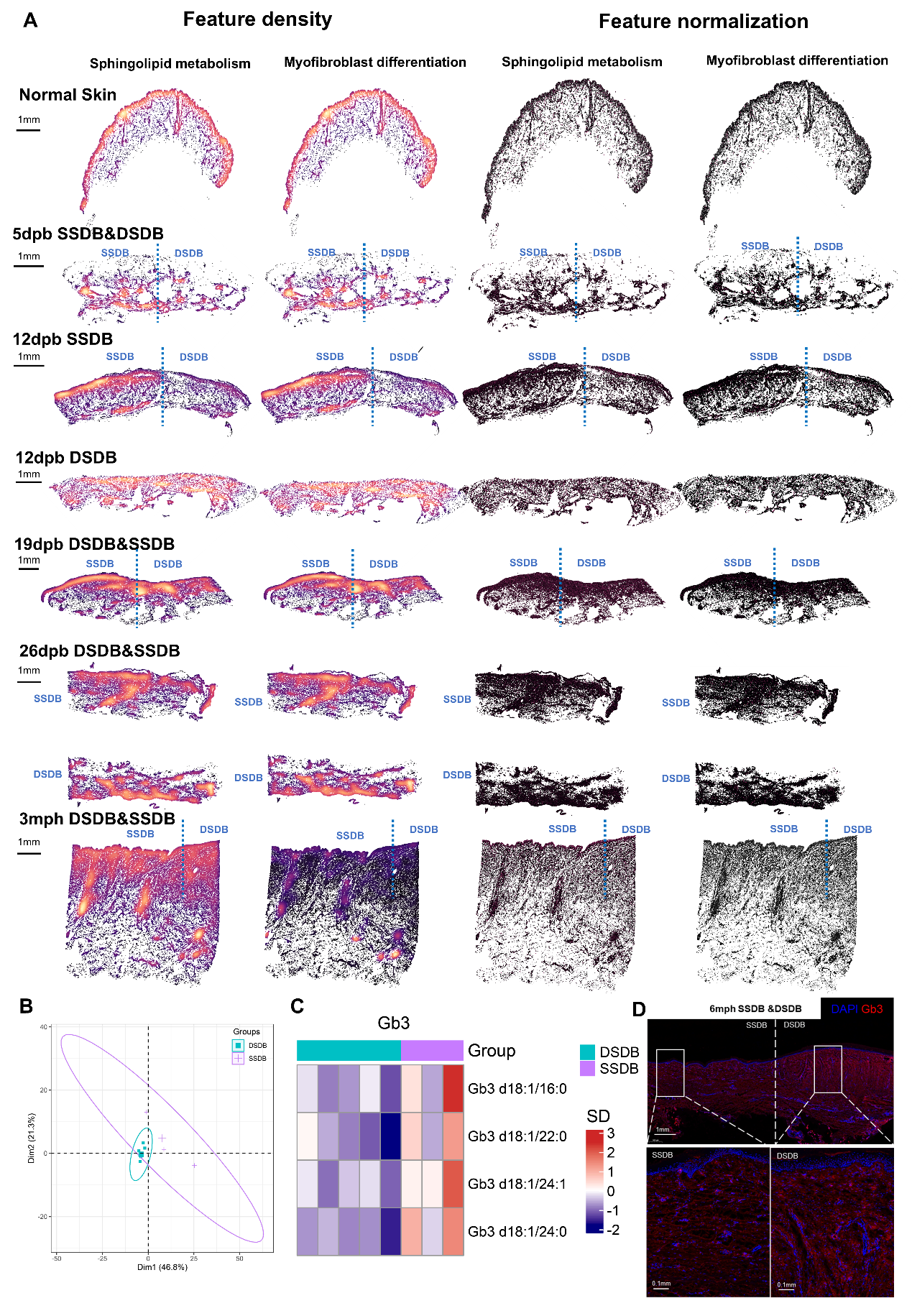


**Supplementary Figure 2 Spatial transcriptome and lipid sequencing sphingolipid expression at different stages of burn**

**A** Feature plots showed sphingolipid metabolism and myofibroblast differentiation pathway expression patterns at each stage after burn. **B** Principal component analysis showed the distribution of SSDB and DSDB samples. **C** Heat map showed the distribution of SSDB and DSDB samples. **D** Immunofluorescence suggested the expression of Gb3 is higher in SSDB than in DSDB.


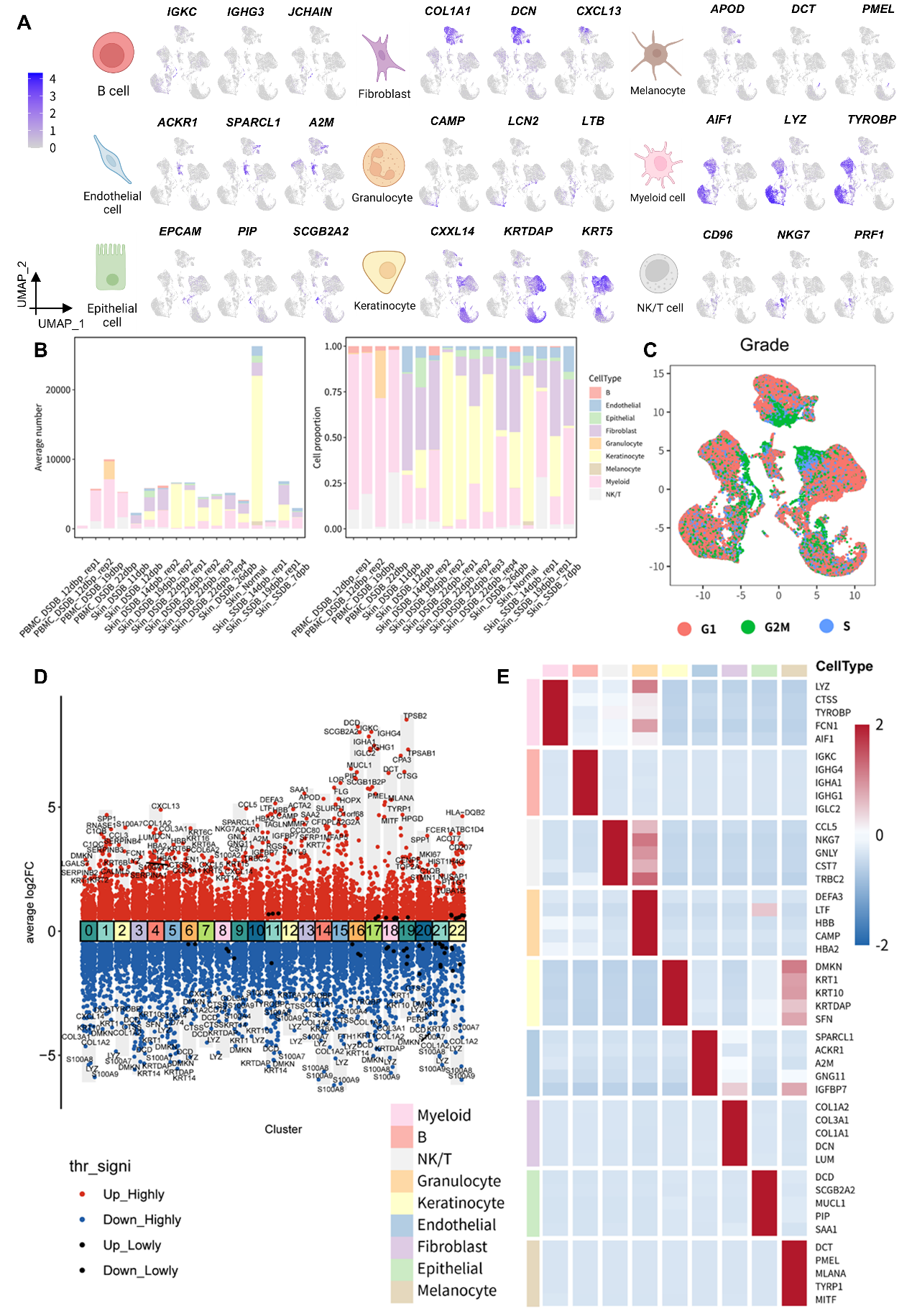


**Supplementary Figure 3 The DEGs landscape of nine cell types identified in scRNA-seq.**

**A** The feature plots of marker genes in nine cell types. **B** The average number (left) and proportion (right) of nine cell types in all samples. **C** Cell cycle analysis showed the distribution of cells in G1-phase, G2M-phase, and S-phase in the UMAP plot. **D** The significant upregulated (red) and downregulated (blue) DEGs among 23 clusters. Each dot represented a DEG. When the average log_2_FC was greater than 0 and adjusted p-value was less than 0.01, the gene was defined as a highly upregulated DEG. When the average log_2_FC was smaller than 0 and adjusted p-value was less than 0.01, the gene was defined as a highly downregulated DEG. **E** The top five expressed DEGs in nine cell types by heatmap.


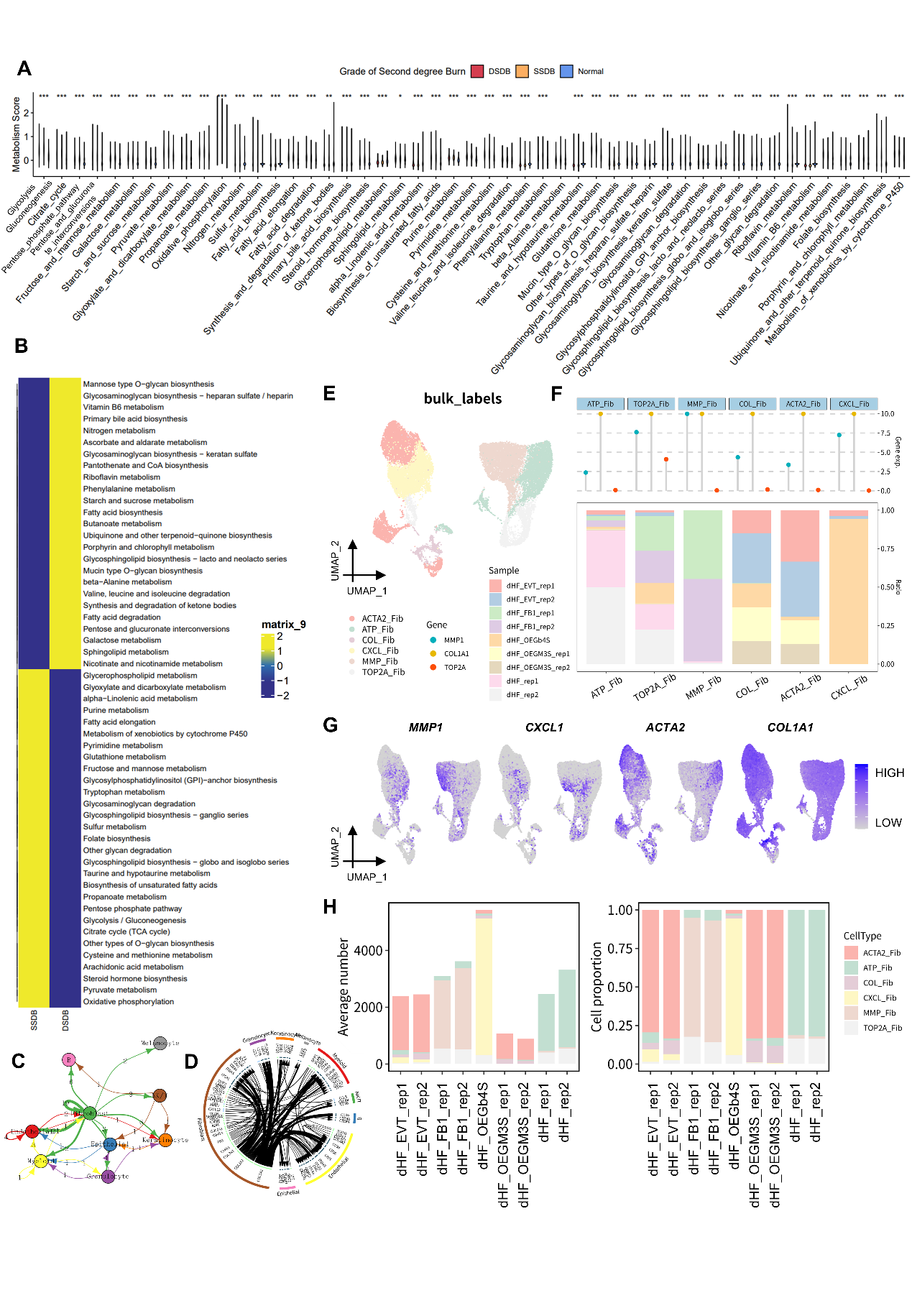


**Supplementary Figure 4 Metabolic pathways differentially express between SSDB and DSDB, and alteration of Gb and GM leads to dHFB heterogeneity.**

**A** GSVA analysis quantitatively showed differential activities of 49 metabolic pathways in DSDB, SSDB, and normal skin at single cell level. **B** The differential expression of 49 metabolic pathways between DSDB and SSDB by heatmap. **C** The interaction network of nine cell types by cell communication analysis. **D** The interactive ligand-receptor pairs among nine cell types by cell communication analysis. **E** The scRNA-seq of five differently treated dHFB groups and identified six fibroblast subtypes. **F** Cleveland plot displayed the distribution of six fibroblast subtypes in nine samples, and typical markers (*MMP1, COL1A1, TOP2A*) expression in each subtype. **G** The feature plot of marker genes, showing their distribution in different fibroblast subtypes. **H** The average number (left) and proportion (right) of six fibroblast subtypes in nine samples.


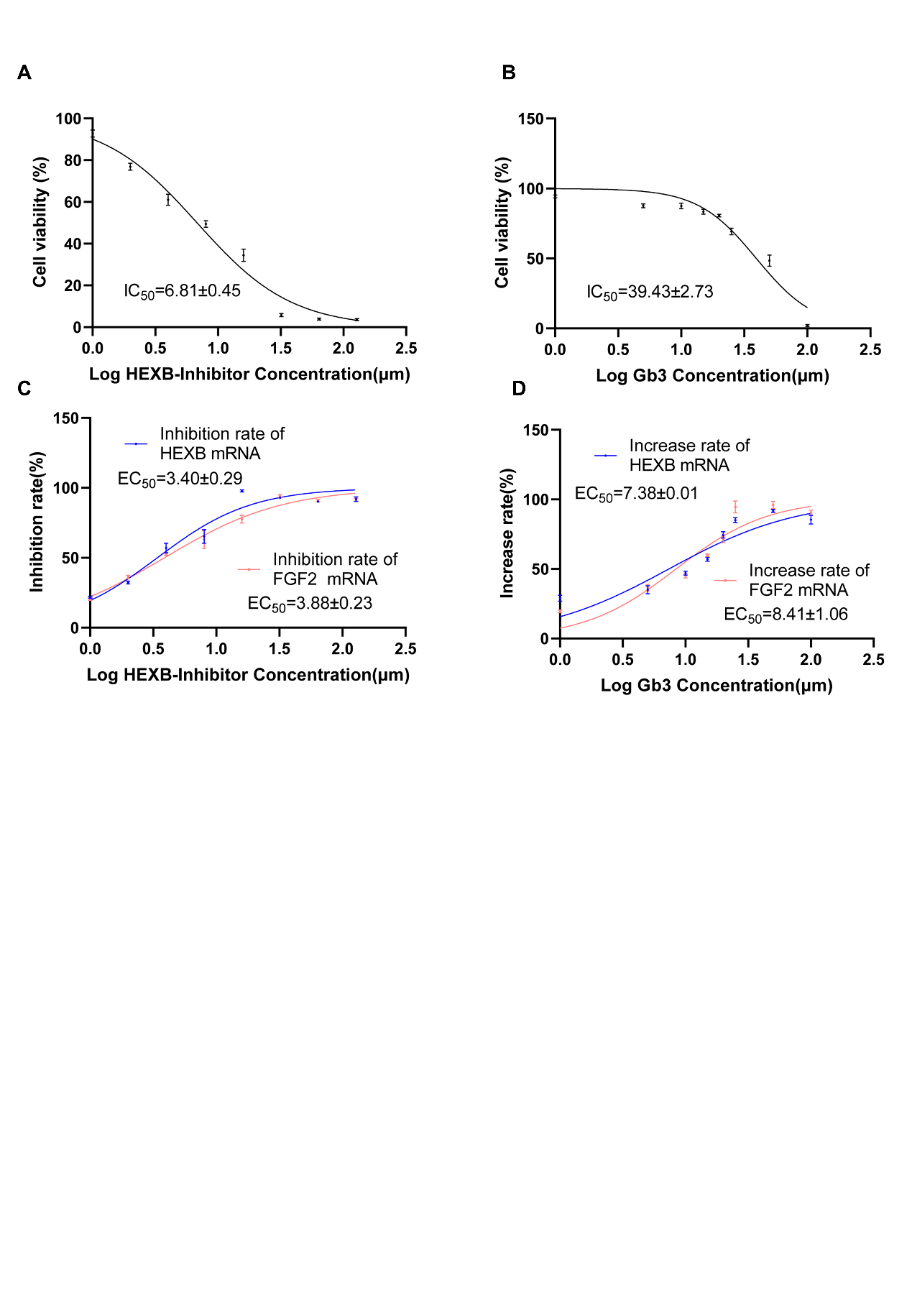


**Supplementary Figure 5 Assessment of Drug Toxicity and Dose-Effect Relationships for HEXB Inhibitor and Gb3.**

A Cytotoxicity assay of cell growth inhibition activity for HEXB-Inhibitor against dHFBs. B Cytotoxicity assay of cell growth inhibition activity for Gb3 against dHFBs. C Dose-response analysis of HEXB and FGF2 inhibition activity for HEXB-Inhibitor against dHFBs. D Dose-response analysis of HEXB and FGF2 increase activity for Gb3 against dHFBs. The data are presented as the means ± SDs.


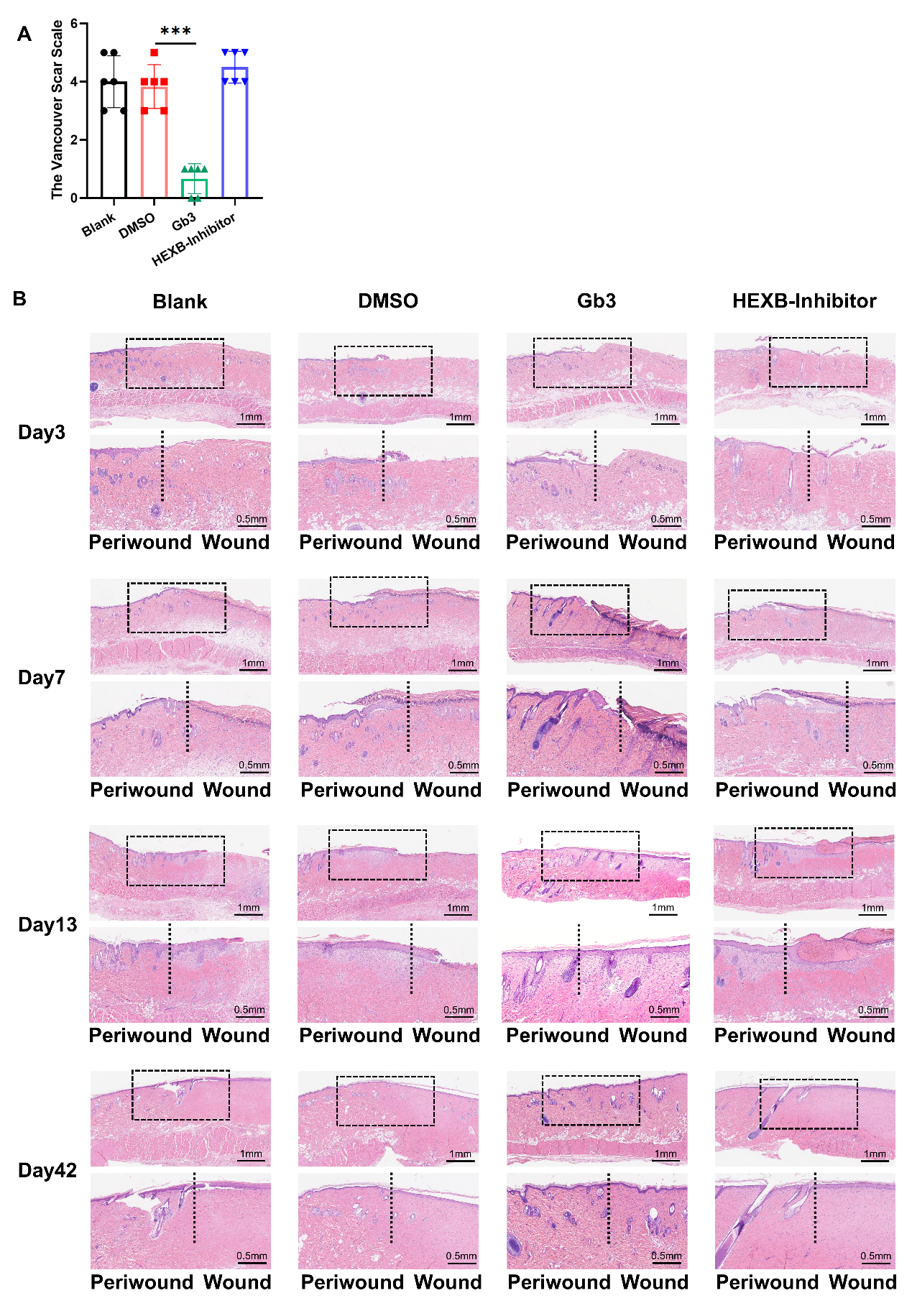


**Supplementary Figure 6 Gb3 accelerates wound closure and improves the quality of healing.**

**A** The Vancouver Scar Scale of animal experiment. (n=6) **B** H&E staining of the wound indicated the healing condition at 3, 7, 13, and 42 days. The data are presented as the means ± SDs. ****p* < 0.001.


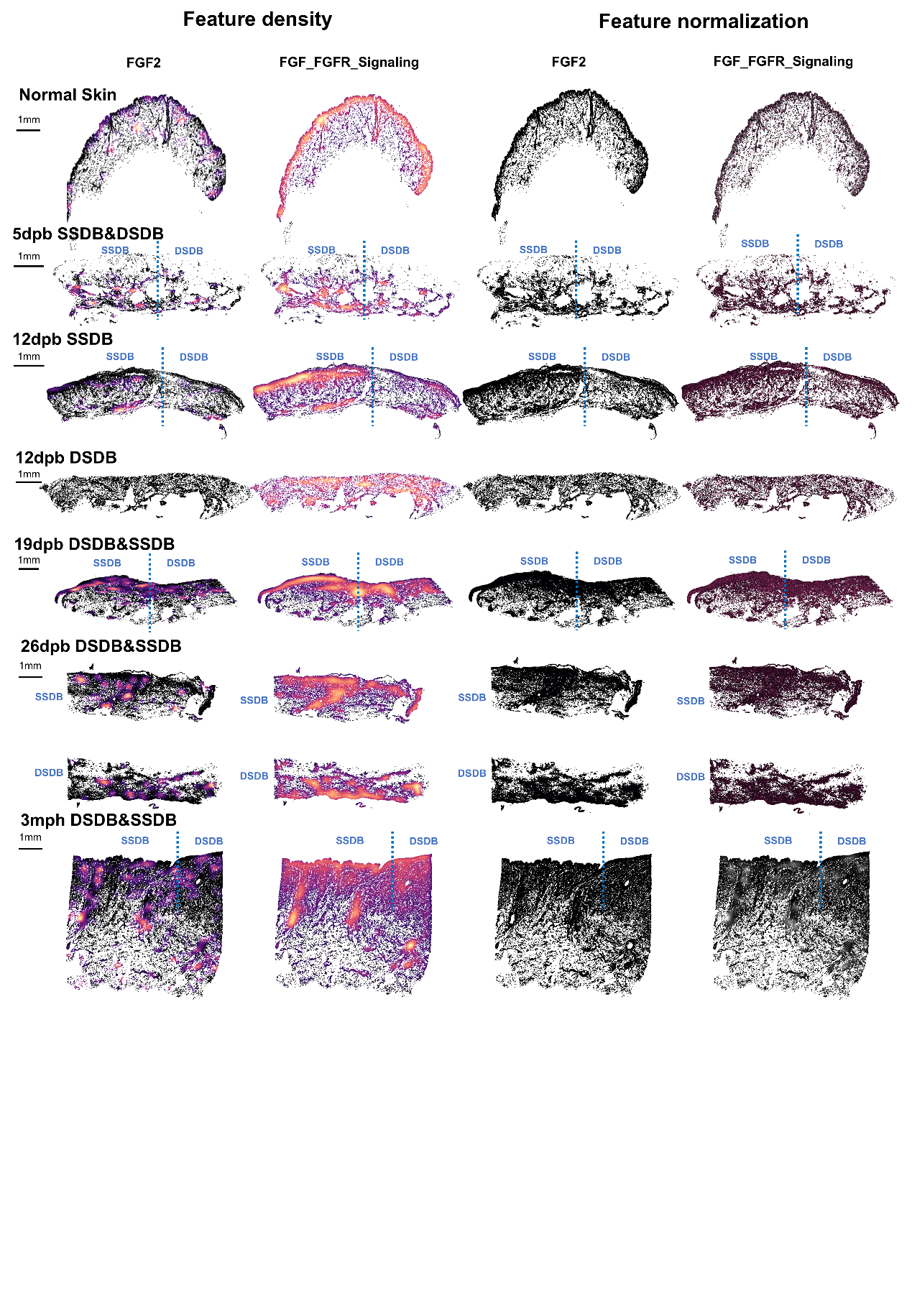


**Supplementary Figure 7 The expression of FGF2 and FGFR pathways in clinical samples.**


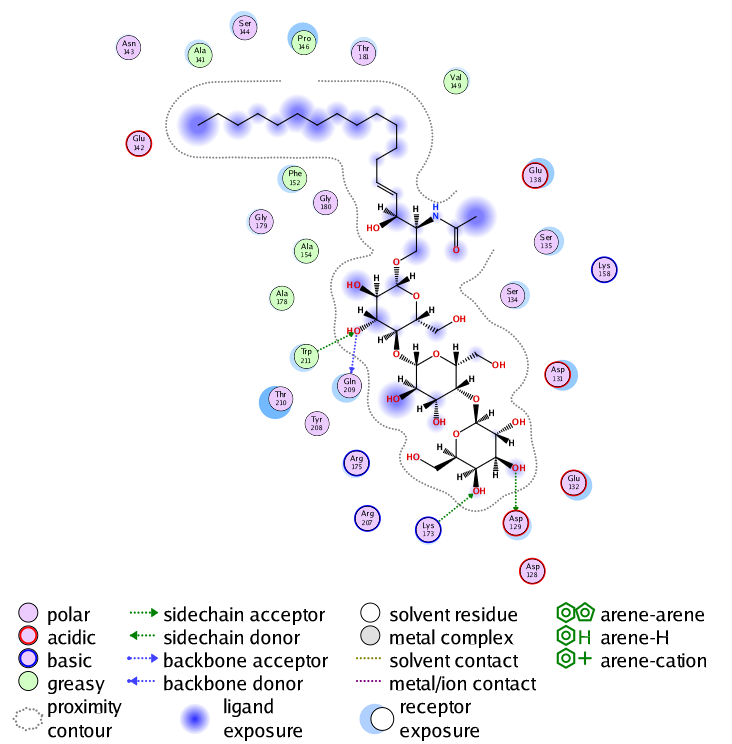


**Supplementary Figure 8 Interaction between Gb3 and FGFR1.**


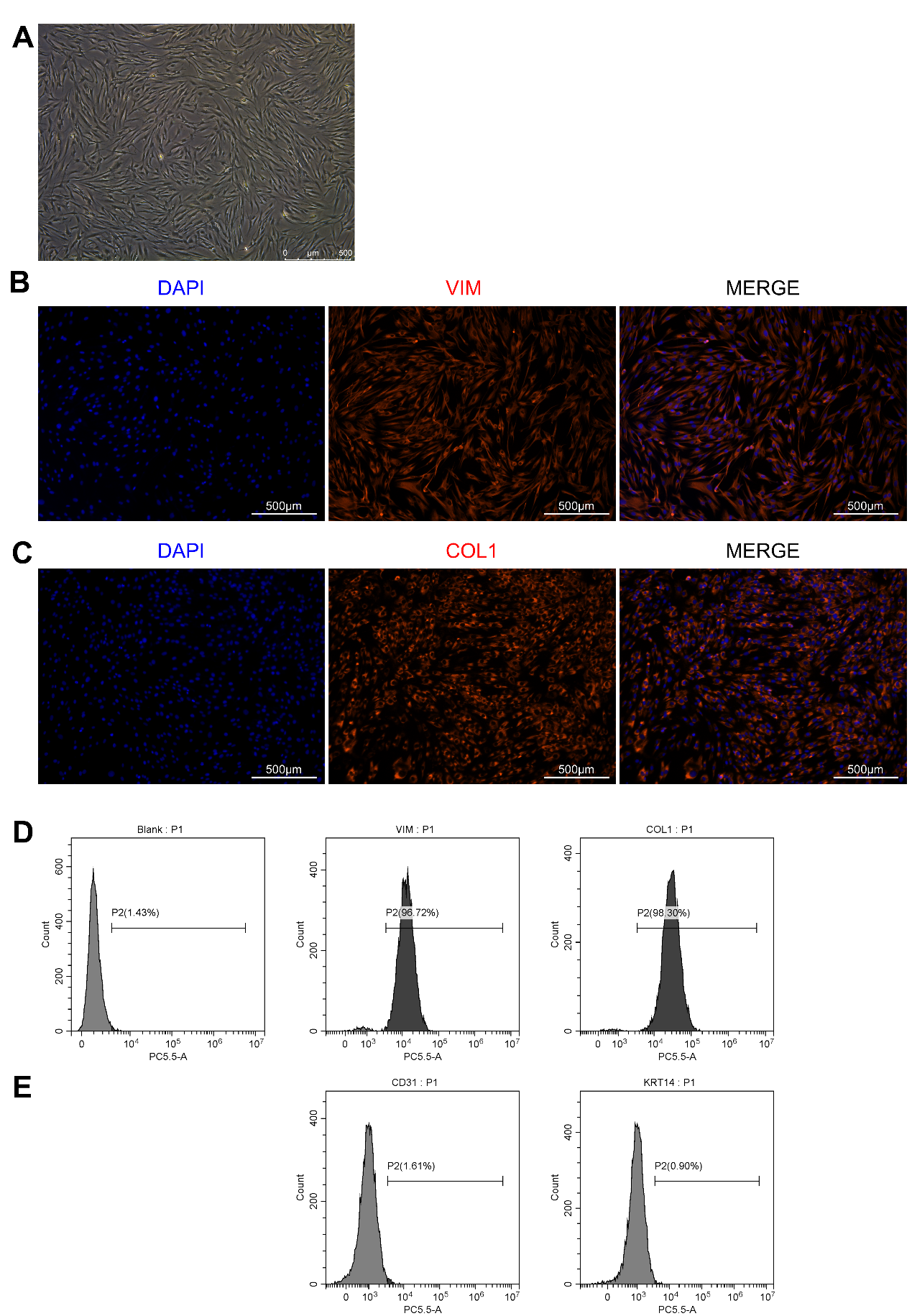


**Supplementary Figure 9 Characterization of primary human dermal fibroblasts (dHFBs)**
**A** Phase-contrast micrograph depicting the typical spindle-shaped morphology of dHFBs.
**B, C** Immunofluorescence staining confirming >95% of dHFBs co-express mesenchymal markers: **(B)** Vimentin (VIM, red) and **(C)** Collagen I (COL1, red). Nuclei counterstained with DAPI (blue).
**D, E** Flow cytometric quantification validating high positivity for VIM (96.72%) and COL1 (98.30%), **(E)** with negligible contamination by endothelial cells (CD31; 1.61%) or keratinocytes (KRT14; 0.90%).


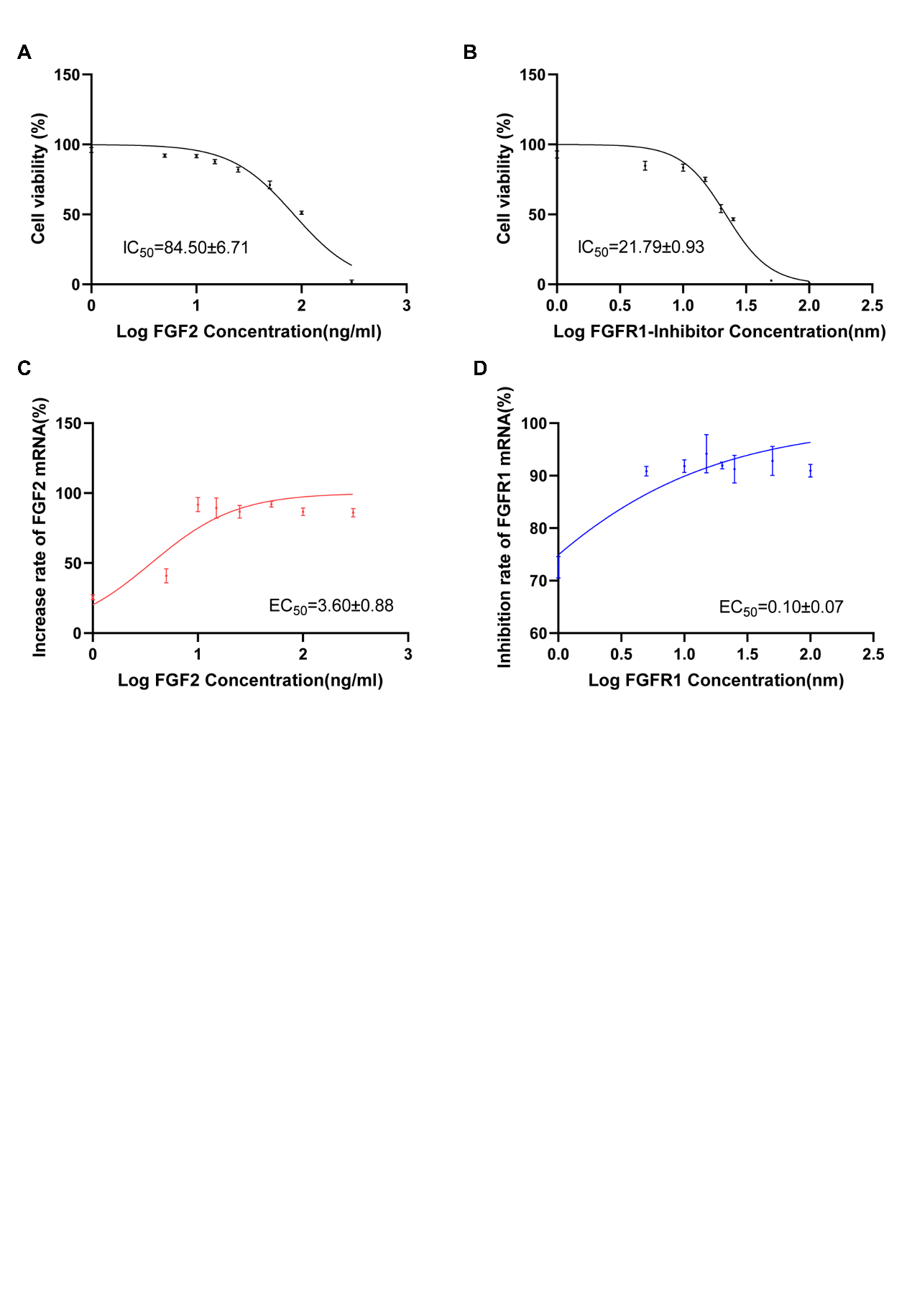


**Supplementary Figure 10 Assessment of Drug Toxicity and Dose-Effect Relationships for FGF2 and FGFR1 Inhibitor.**

A Cytotoxicity assay of cell growth inhibition activity for FGF2 against dHFBs. B Cytotoxicity assay of cell growth inhibition activity for FGFR1-Inhibitor against dHFBs. C Dose-response analysis of FGF2 increase activity for FGF2 against dHFBs. D Dose-response analysis of HEXB and FGFR1 inhibition activity for FGFR1-Inhibitor against dHFBs. The data are presented as the means ± SDs.

# Tables

**Supplementary Table 1 Clinical characteristics of patients of lipidomic analysis.**

| Patient ID | Sample ID | Grade | Gender | Age | Tissue | Sequencing |
| --- | --- | --- | --- | --- | --- | --- |
| Patient1 | SSDB_Skin1 | SSDB | Male | 40 | Skin | Lipidomics-seq |
|  | DSDB_Skin1 | DSDB | Male | 40 | Skin | Lipidomics-seq |
| Patient2 | SSDB_Skin2 | SSDB | Male | 37 | Skin | Lipidomics-seq |
|  | DSDB_Skin2 | DSDB | Male | 37 | Skin | Lipidomics-seq |
| Patient3 | SSDB_Skin3 | SSDB | Male | 28 | Skin | Lipidomics-seq |
|  | DSDB_Skin3 | DSDB | Male | 28 | Skin | Lipidomics-seq |
| Patient4 | DSDB_Skin4 | DSDB | Male | 47 | Skin | Lipidomics-seq |
| Patient5 | DSDB_Skin5 | DSDB | Male | 35 | Skin | Lipidomics-seq |

Abbreviation: SSDB, superficial second-degree burn; DSDB, deep second-degree burn.

**Supplementary Table 2 The Vancouver Scar Scale.**

|  | Scar characteristic | Score |
| --- | --- | --- |
| Vascularity | Normal | 0 |
|  | Pink | 1 |
|  | Red | 2 |
|  | Purple | 3 |
| Pigmentation | Normal | 0 |
|  | Hypopigmentation | 1 |
|  | Hyperpigmentation | 2 |
| Pliability | Normal | 0 |
|  | Supple | 1 |
|  | Yielding | 2 |
|  | Firm | 3 |
|  | Ropes | 4 |
|  | Contracture | 5 |
| Height | Flat | 0 |
|  | <2 mm | 1 |
|  | 2-5 mm | 2 |
|  | >5 mm | 3 |
|  | **Total score** | **13** |

**Supplementary Table 3 Characterization of FGFR1-Gb3 Interaction**

| Ligand atom | Atomic name | Protein residue | Residue number | Interaction | Distance (Å) | Energy (kcal/mol) |
| --- | --- | --- | --- | --- | --- | --- |
| O8 | O | GLN | 209 | H-donor | 2.94 | -1.8 |
| O12 | OD1 | ASP | 129 | H-donor | 2.68 | -2.7 |
| O8 | NE1 | TRP | 211 | H-acceptor | 2.95 | -1.3 |
| O13 | NZ | LYS | 173 | H-acceptor | 3.36 | -5.2 |

Abbreviation: GLN, Glutamine; ASP, Aspartic acid; TRP, Tryptophan; LYS, Lysine.

**Supplementary Table 4 Baseline clinical metadata of all 24 samples obtained from 6 patients.**

| Patient ID | Sample ID | Grade | Gender | Age | Time | Tissue | Sequencing |
| --- | --- | --- | --- | --- | --- | --- | --- |
| Patient1 | Skin_SSDB_7dpb | SSDB | Male | 53 | 7dpb | Skin | scRNA-seq |
|  |  |  |  |  |  |  |  |
|  | Skin_DSDB_11dpb | DSDB | Male | 53 | 11dpb | Skin | scRNA-seq |
|  |  |  |  |  |  |  |  |
|  | Skin_SSDB_14dpb_rep1 | SSDB | Male | 53 | 14dpb | Skin | scRNA-seq |
|  |  |  |  |  |  |  |  |
|  | Skin_DSDB_14dpb_rep2 | DSDB | Male | 53 | 14dpb | Skin | scRNA-seq |
|  |  |  |  |  |  |  |  |
|  | Skin_Normal | HNS | Male | 53 | HNS | Skin | scRNA-seq |
|  |  |  |  |  |  |  |  |
|  | Skin_DSDB_22dpb_rep1 | DSDB | Male | 53 | 22dpb | Skin | scRNA-seq |
| Patient2 | Skin_DSDB_12dpb | DSDB | Female | 49 | 12dpb | Skin | scRNA-seq |
|  |  |  |  |  |  |  |  |
|  | Skin_SSDB_19dpb_rep1 | SSDB | Female | 49 | 19dpb | Skin | scRNA-seq |
|  |  |  |  |  |  |  |  |
|  | Skin_DSDB_19dpb_rep2 | DSDB | Female | 49 | 19dpb | Skin | scRNA-seq |
|  | Skin_DSDB_22dpb_rep2 | DSDB | Female | 49 | 22dpb | Skin | scRNA-seq |
|  | Skin_DSDB_26dpb | DSDB | Female | 49 | 26dpb | Skin | scRNA-seq |
| Patient3 | Skin_DSDB_22dpb_rep3 | DSDB | Male | 69 | 22dpb | Skin | scRNA-seq |
| Patient4 | Skin_SSDB & DSDB_5dpb | SSDB & DSDB | Male | 44 | 5dpb | Skin | Stereo-seq |
|  | Skin_SSDB_12dpb | SSDB | Male | 44 | 12dpb | Skin | Stereo-seq |
|  | Skin_DSDB_12dpb | DSDB | Male | 44 | 12dpb | Skin | Stereo-seq |
|  | Skin_SSDB & DSDB_19dpb_rep1 | SSDB & DSDB | Male | 44 | 19dpb | Skin | Stereo-seq |
|  | Skin_DSDB_22dpb_rep4 | DSDB | Male | 44 | 22dpb | Skin | scRNA-seq |
|  | Skin_SSDB & DSDB_26dpb | SSDB & DSDB | Male | 44 | 25dpb | Skin | Stereo-seq |
|  | PBMC_DSDB_12dpb_rep1 | DSDB | Male | 44 | 12dpb | PBMC | scRNA-seq |
|  | PBMC_DSDB_19dpb | DSDB | Male | 44 | 19dpb | PBMC | scRNA-seq |
|  | PBMC_DSDB_22dpb | DSDB | Male | 44 | 22dpb | PBMC | scRNA-seq |
| Patient5 | PBMC_DSDB_12dpb_rep2 | DSDB | Male | 33 | 12dpb | PBMC | scRNA-seq |
|  | Skin_SSDB & DSDB_19dpb_rep2 | SSDB & DSDB | Male | 33 | 19dpb | Skin | ST (FFPE) |
| PATIENT6 | Skin_SSDB & DSDB_6mph | SSDB & DSDB | Male | 54 | 6mph | Skin | Stereo-seq |
|  | Skin_SSDB & DSDB_3mph | SSDB & DSDB | Male | 54 | 3mph | Skin | Stereo-seq |

Abbreviation: Stereo-seq, Spatial Enhanced Resolution Omics-sequencing; ST, 10x Visium spatial transcriptome; FPEE, Formalin-fixed, Paraffin-embedded; dpb, day post-burn; mph, months post-healing; SSDB, superficial second-degree burn; DSDB, deep second-degree burn.

**Supplementary Table 5 List of antibodies used in this study.**

| **Antibodies** | **SOURCE** | **IDENTIFIER** |
| --- | --- | --- |
| **HEXB** | Santa Cruz | Cat#sc-376781; RRID: AB_2909474 |
| **ACTA2** | Abcam | Cat#ab7817; RRID: AB_262054 |
| **MMP1** | CST | Cat#E9S9N; RRID: AB_2799459 |
| **FGF2** | Santa Cruz | Cat#sc-74412; RRID: AB_1122854 |
| **GAPDH** | Epizyme Biotech | Cat#LF206; RRID: AB_3697174 |
| **Anti-Mouse IgG-HRP** | Epizyme Biotech | Cat#LF101; RRID: AB_3083706 |
| **Anti-Rabbit IgG-HRP** | Epizyme Biotech | Cat#LF102; RRID: AB_3083707 |
| **HEXB** | Proteintech | Cat#16229-1-AP; RRID: AB_2264075 |
| **FAP** | Abclone | Cat#A23789; RRID: AB_3492061 |
| **CD90** | Santa Cruz | Cat#sc-53456; RRID: AB_630308 |
| **Gb3** | GeneTex | Cat#GTX30743; RRID: AB_1240607 |
| **FGFR1** | CST | Cat#D8E4; RRID: AB_2797940 |
| **VIM** | CST | Cat#D21H3; RRID: AB_10695149 |
| **COL1** | abcam | Cat#ab138492; RRID: AB_2861258 |
| **KRT14** | abcam | Cat#ab119695; RRID: AB_10898957 |
| **CD31** | abcam | Cat#ab24590; RRID: AB_448167 |
| **Goat anti-rabbit Alexa594** | Invitrogen | Cat#A11012; RRID: AB_2534079 |
| **Goat anti-rabbit Alexa488** | Invitrogen | Cat#A11008; RRID: AB_143165 |
| **Goat anti-mouse Alexa594** | Invitrogen | Cat#A11032; RRID: AB_2534091 |
| **Goat anti-rat Alexa488** | Invitrogen | Cat#A48262TR; RRID: AB_2896331 |

**Supplementary Table 6 List of Quantitative Real-time primers used in this study.**

| **Human qRT-PCR Primers** | | |
| --- | --- | --- |
| **Gene Symbol** | **Primer Sequences (5‘– 3’)** | |
| ***18S*** | Forward | GTAACCCGTTGAACCCCATT |
|  | Reverse | CCATCCAATCGGTAGTAGCG |
| ***ACTA2*** | Forward | ACTGCCTTGGTGTGTGACAA |
|  | Reverse | CACCATCACCCCCTGATGTC |
| ***COL1*** | Forward | CATGGAGACTGGTGAGACCT |
|  | Reverse | GCCATACTCGAACTGGAATC |
| ***CCRL1*** | Forward | TGAGGGTCCTACAGAGCCAACCA |
|  | Reverse | CTCCCCCTTCCCCCAACCCA |
| ***CXCL5*** | Forward | TACAGACCACGCAAGGAGTT |
|  | Reverse | TAGAAAAGGGGCTTCTGGAT |
| ***FGF2*** | Forward | GCGACCCTCACATCAAGCTA |
|  | Reverse | AGCCAGGTAACGGTTAGCAC |
| ***HEXB*** | Forward | TCCAAGATTATGGCCTCGGG |
|  | Reverse | GGTTGTGCAGCTATTCCACG |
| ***HGF*** | Forward | CACGGAAGAGGAGATGAGAA |
|  | Reverse | TTTTCAGGAATTGTGCATCC |
| ***LMNA*** | Forward | GGAACTCTGAGGGCTGCAAT |
|  | Reverse | GAGTTCAGCAGAGCCTCCAG |
| ***MGP*** | Forward | TTTGTGTTATGAATCACATGAAAGC |
|  | Reverse | AGCGTTCTCGGATCCTCTCT |
| ***MMP1*** | Forward | ACACCTCTGACATTCACCAAG |
|  | Reverse | ATGAGCCGCAACACGATG |
| ***PDPN*** | Forward | GGGAAGGTACTCGCCCTAAA |
|  | Reverse | GGTCATCTTCTCCCACGAGC |
| ***VIM*** | Forward | GGCGAGGAGAGCAGGATTTC |
|  | Reverse | TGGGTATCAACCAGAGGGAGT |
| ***FGFR1*** | Forward | ACCACCGACAAAGAGATGGA |
|  | Reverse | GCAGAGTGATGGGAGAGTCC |
| ***FGFR2*** | Forward | GATAAATAGTTCCAATGCAGAAGTGCT |
|  | Reverse | TGCCCTATATAATTGGAGACCTTACA |
| ***FGFR3*** | Forward | CAAGTTTGGCAGCATCCGGCAGAC |
|  | Reverse | CACCACCAGCCACGCAGAGTGATG |
| ***FGFR4*** | Forward | GAGGGGCCGCCTAGAGATT |
|  | Reverse | CAGGACGATCATGGAGCCT |

# Files
