# Supplementary material for: Globotriaosylceramide Gb3 Influences Wound Healing and Scar Formation by Orchestrating Fibroblast Heterogeneity
Source: Adv Sci (Weinh). 2025 Aug 14;12(41):e09733. doi: 10.1002/advs.202509733 (PMC12591104; doi:10.1002/advs.202509733)
Supplement: Supplementary file 2 — Supporting Information [file ADVS-12-e09733-s001.zip › advs71303-sup-0002-Data/Supplementary File1.pdf]

上海翼和应用生物技术有限公司
SHANGHAI BIOWING APPLIED BIOTECHNOLOGY CO.LTD

Mycoplasma Test Report

Sample Information

**Report ID**	20250530_1
**Sample Name**	dHFB
**Sample from**	Changhai hospital
**Test Item**	Detection of Mycoplasma
**Testing Method:**	Fluorescence Quantitative PCR

Introduction

☆ Based on fluorescence quantitative PCR method, mycoplasma DNA can be detected with a probe labeled with FAM fluorescence in a single reaction.

☆ To avoiding false negative, inner control of probe is labeled with VIC fluorescence to monitoring experiments.

☆ To avoiding false positive, a close-tube operation and a negative control are used in entire amplification.

☆ Fluorescence quantitative PCR is highly sensitive with detection as few as 10 copies of mycoplasma DNA.

Quality control

1、The positive standard product detection channel (FAM), internal standard channel (HEX) showed a standard "S" type amplification curve, positive quality control was established.

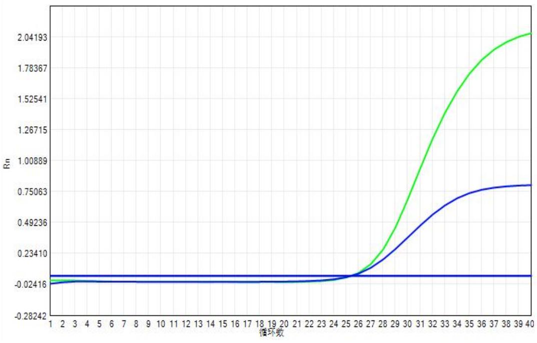

2、The Negative reference detection channel (FAM) showed no amplification, internal standard channel (HEX) showed standard "S" type amplification curve, and negative quality control was established.

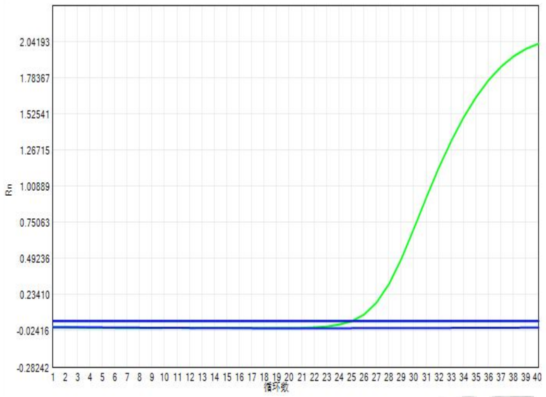

Detection Result

1、The Sample amplification curve of detection channel (FAM) (left) and internal standard channel (HEX) (right). The internal standard channel showed an "S" amplification curve, indicating that the PCR process of the sample was normal.

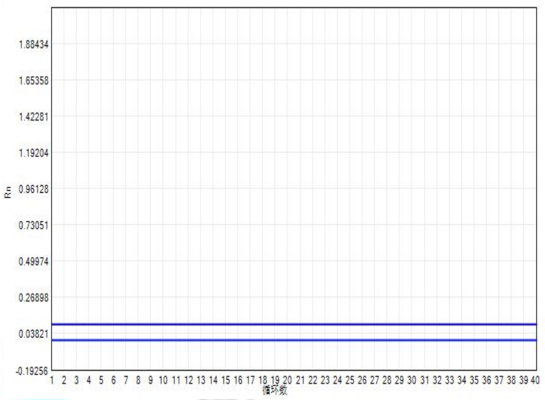

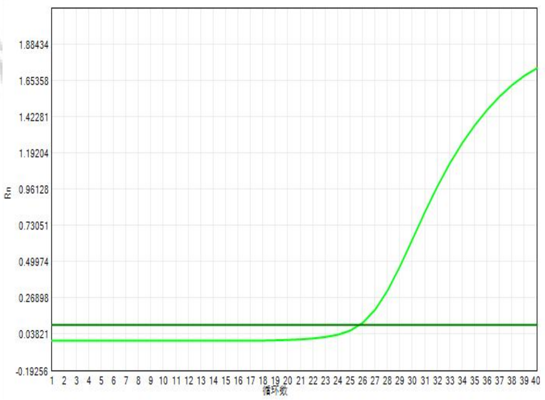

2、Qualitative standards

Detection channel (FAM) Ct value range	Internal standard channel (HEX) Ct range	Qualitative Result
Ct≤19	Ct≤29	Strong Positive
19<Ct≤25	Ct≤29	Positive
25<Ct≤30	Ct≤29	Weak Positive
Ct>33	Ct≤29	Negative
30<CT≤33	Ct≤29	Gray zone, requiring verification

3、Summary of test results

Test sample\channel	Test sample Ct	Inner Standard Ct	Qualitative Result	Reference concentration
Positive standard	Ct∈(23-29)	25.85	-	-
Negative standard	NoCt	25.45	-	-
Tested sample	NoCt	25.71	Negative	-

Conclusion

1、The amplification results of positive reference were normal, and the Ct values of each reference were linear. The negative reference detection channel showed no amplification, the internal standard gene was normally amplified, the quality control link was qualified, and the experiment process was not abnormal.

2、The internal standard gene amplification of the tested sample was normal, and the Ct value was 25.71, indicating that the PCR process of the tested product was normal.

3、The Ct value of the detection channel of the tested sample is NoCt, and the test result of your sample mycoplasma is: positive.

Inspector： Chenqian Zhang
Reviewer： Kaiyue Chao
Support hotline： 0510-85996869
Date of issue： 20250530
