# Supplementary material for: Globotriaosylceramide Gb3 Influences Wound Healing and Scar Formation by Orchestrating Fibroblast Heterogeneity
Source: Adv Sci (Weinh). 2025 Aug 14;12(41):e09733. doi: 10.1002/advs.202509733 (PMC12591104; doi:10.1002/advs.202509733)
Supplement: Supplementary file 2 — Supporting Information [file ADVS-12-e09733-s001.zip › advs71303-sup-0002-Data/Supplementary File2.pdf]

Cell Line Authentication Service

STR Profiling Report

Sample From: Changhai hospital

Sample Type: Cell Line

Testing Method: STR Genotyping

Report Time: June 03 2025

Add: Room 518, No.2, Zhongxing Creative Park, Lane 1015, Longteng Road, Songjiang District, Shang[illegible]
/ Tel:+86-021-33559491

COMPANY STATEMENT

1. THIS REPORT IS ONLY RESPONSIBLE FOR THE SAMPLES ANALYZED.
2. THE TESTING RESULTS AND THE ORGANIZATION NAME WILL NOT BE USED FOR ADVERTISEMENT, COMMERCIAL EXHIBITIONS, COMMERCIAL PERFORMANCE AND OTHER COMMERCIAL ACTIVITIES.
3. OBJECTIONS SHOULD BE RAISED WITHIN FIFTEEN DAYS AFTER THE RECEIPT OF THIS REPORT.
4. THE PAPER REPORT WITH CONTENT ALTERING, ADDING OR WITHOUT THE STAMPED SEAL OF THE COMPANY ARE INVALID.

Testing Company: Shanghai Biowing Applied Biotechnology Co. Ltd

Address: Room 502, No.2, Zhongxing Creative Park, Lane 1015, Longteng Road, Songjiang District, Shanghai **Tel:** +86-021-33559491

Contact: Wenyao Zhang

E-mail:market@biowing.com.cn

Cell Line Authentication – STR Profiling Report

Sample code

Table 1. Sample Code

Customer's code	Company Code
dHFB	20250530-01

Sample Number:1

Sample Type: Cell line

Testing Type: STR

Testing Method:

DNA was extracted by a commercial kit from CORNING (AP-EMN-BL-GDNA-250G). The twenty STRs including Amelogenin locus were amplified by six multiplex PCR and separated on ABI 3730XL Genetic Analyzer. The signals were then analyzed by the software GeneMapper.

Data Interpretation:

Cell lines were authenticated using Short Tandem Repeat (STR) analysis asdescribed in 2012 in ANSI Standard (ASN-0002) by the ATCC Standards Development Organization (SDO) and in Capes-Davis et al., Match criteria for human cell line authentication: Where do we draw the line? Int J Cancer.2013;132(11):2510-9.

Add: Room 518, No.2, Zhongxing Creative Park, Lane 1015, Longteng Road, Songjiang District, Shang
/ Tel:+86-021-33559491

Test Results

(一) Inspect the basic situation

Table 2. Sample genotype test results

Company Code	Polyallele	Matching cell line	Cell bank	EV	Matching instructions
20250530-01	No		EXPASY		No match

- Polyalleles refer to the phenomenon of genes at the third isoposition and above.
- The typing results of each cell in this test were good.

(二) Description of each sample

● 20250530-01: The DNA typing of this strain of cells did **not find a matching cell line** in the cell line search. **No polyalleles** were found in this cell line in this test.

Remarks: The cell lines to be tested were included in ATCC and DSMZ (DSMZ included STR data of 2,490 cell lines from ATCC, DSMZ, JCRB and RIKEN, etc.). STR data in the ExPASy cell bank (ExPASy contains STR data of approximately 8,000 human cell strains from datasets such as ATCC, DSMZ, JCRB, ECACC and Riken) are matched. Cells not included in the above-mentioned cell banks will not be matched. According to the identification standard of the ATCC Standards Committee (ANSI/ATCC ASN-0002-2022), a matching degree EV of ≥80% is considered to be correlated and may have originated from common ancestral cells. The matching degree is between 55% and 80%, and it is necessary to further identify and verify its correlation in combination with other methods.

Add: Room 518, No.2, Zhongxing Creative Park, Lane 1015, Longteng Road, Songjiang District, Shang
/ Tel:+86-021-33559491

Test Results

1. STR profile

Table 3. STR and Amelogenin Genotyping Results of Cell line 20250530-01.

Loci	Sample information			Cell Bank information		
	Sample name: dHFB			Cell line name:		
	**Allele1**	**Allele2**	**Allele3**	**Allele1**	**Allele2**	**Allele3**
D5S818	11	12				
D13S317	8	9				
D7S820	8	11				
D16S539	9	10				
VWA	17	17				
TH01	6	7				
AMEL	X	Y				
TPOX	8	8				
CSF1PO	11	12				
D12S391	20	20				
FGA	19	21				
D2S1338	24	25				
D21S11	29	31				
D18S51	13	14				
D8S1179	13	13				
D3S1358	15	17				
D6S1043	13	19				
PENTAE	11	12				
D19S433	14	16				
PENTAD	9	11				
D1S1656	12	17				

Add: Room 518, No.2, Zhongxing Creative Park, Lane 1015, Longteng Road, Songjiang District, Shang
/ Tel:+86-021-33559491

2. database annotation

Table 4. STR matching analysis

Accession	Name	Nº Markers	Score	Amel	CSF1PO	D1S1656	D2S1338	D3S1358	D5S818	D6S1043	D7S820	D8S1179	D12S391	D13S317	D16S539	D18S51	D19S433	D21S11	FGA	PentaD	PentaE	TH01	TPOX	vWA
**NA**	**Query**	**NA**	**NA**	**X,Y**	**11,12**	**12,17**	**24,25**	**15,17**	**11,12**	**13,19**	**8,11**	**13**	**20**	**8,9**	**9,10**	**13,14**	**14,16**	**29,31**	**19,21**	**9,11**	**11,12**	**6,7**	**8**	**17**
CVCL_8305	PL504	8	80.00%	X	12				11		8,11			8,9	9,10							9	8	17
CVCL_Y018	HA59T/VGH	8	78.57%	X,Y	11,12				10,11,12		8,11			9	9,10							9	8	17,21

Note: The STR online match analysis of the test cell against EXPASY database, showing cell number (Cell No.) and cell name.

3. Authentication

☑ The submitted sample profile is human, but not a match for any profile in the DSMZ and EXPASY STR database.

☐ The submitted profile is an exact match for the following human cell line(s) in the EXPASY STR database (8 core loci plus Amelogenin):

☐ The submitted profile is similar to the following DSMZ human cell line:

- **Note:** A cell line can be considered to be authenticated when more than 80% of the alleles in its STR profile match profiles from tissue or other cell line samples from that donor or from database. Cell lines with between a 55% to 80% match require further profiling for investigation of relatedness.

Add: Room 518, No.2, Zhongxing Creative Park, Lane 1015, Longteng Road, Songjiang District, Shang
/ Tel:+86-021-33559491

Appendix:

1. Genotyping Strategy and Site Distribution

Table S1. Experimental Strategy and Sites

	**Strategy 1**	**Strategy 2**	**Strategy 3**	**Strategy 4**
1	D3S1358	D8S1179	D19S433	AMEL
2	VWA	D21S11	TH01	D1S1656
3	D7S820	D16S539	D13S317	D5S818
4	CSF1PO	D2S1338	TPOX	D12S391
5	PENTAE	PENTAD	D18S51	FGA
6			D6S1043	

The allele match algorithm compares the 8 core loci plus amelogenin only, even though alleles from all lociwill be reported when available.

2. DSMZ tools was used to carry on the cell line comparison, which contains 2455 cell lines STR data from ATCC, DSMZ, JCRB ,ECACC， GNE and RIKEN databases. If the cell is not included in the above cell library, users need to compared with other databases.

Technician: Xiuchuan He

Checked by: Chengjian Zhang

Issued by: Wang Min

Issue date: June 0[illegible]25

Add: Room 518, No.2, Zhongxing Creative Park, Lane 1015, Longteng Road, Songjiang District, Shang
/ Tel:+86-021-33559491
